# Robust Portfolio Optimization Using Pseudodistances

**DOI:** 10.1371/journal.pone.0140546

**Published:** 2015-10-15

**Authors:** Aida Toma, Samuela Leoni-Aubin

**Affiliations:** 1 Department of Applied Mathematics, Bucharest Academy of Economic Studies, Bucharest, Romania; 2 “Gh. Mihoc—C. Iacob” Institute of Mathematical Statistics and Applied Mathematics, Romanian Academy, Bucharest, Romania; 3 INSA Lyon, ICJ, Villeurbanne Cedex, France; Universita’ del Piemonte Orientale, ITALY

## Abstract

The presence of outliers in financial asset returns is a frequently occurring phenomenon which may lead to unreliable mean-variance optimized portfolios. This fact is due to the unbounded influence that outliers can have on the mean returns and covariance estimators that are inputs in the optimization procedure. In this paper we present robust estimators of mean and covariance matrix obtained by minimizing an empirical version of a pseudodistance between the assumed model and the true model underlying the data. We prove and discuss theoretical properties of these estimators, such as affine equivariance, B-robustness, asymptotic normality and asymptotic relative efficiency. These estimators can be easily used in place of the classical estimators, thereby providing robust optimized portfolios. A Monte Carlo simulation study and applications to real data show the advantages of the proposed approach. We study both in-sample and out-of-sample performance of the proposed robust portfolios comparing them with some other portfolios known in literature.

## Introduction

Since Markowitz [1] formulated the idea of diversification of investments, the mean-variance approach has been widely used in practice in asset allocation and portfolio management, despite many sophisticated models proposed in literature. On the other hand, some drawbacks of the standard Markowitz approach are reported in literature (see [2]). One of the critical weaknesses of the classical mean-variance analysis is its lack of robustness. Since the classical estimators of the mean and the covariance matrix, which are inputs in the optimization procedure, are very sensitive to the presence of gross errors or atypical events in data, the weights of the resulted portfolio, which are outputs of this procedure, can be drastically affected by these atypical data. This fact was proved in [3] by using the influence function approach. In order to remove this drawback and to construct portfolios not overly affected by deviations of the data from the assumed model, many methods have been proposed in literature. For an overview on the methods used for robust portfolio optimization we refer to [4]. Among the methods which improve the stability of portfolio weights by using robust estimators of the mean and covariance, we recall those proposed by Vaz-de Melo and Camara [5] which use M-estimators, Perret-Gentil and Victoria-Feser [3] which use the translated biweight S-estimator, Welsch and Zhou [6] which use minimum covariance determinant estimator and winsorization, DeMiguel and Nogales [7] which use both M- and S-estimators, Ferrari and Paterlini [8] which use Maximum L_*q*_-Likelihood Estimators. These contributions have the merit to consider the role of robust estimation for improving the mean-variance portfolios. On the other hand, it is known that traditional robust estimators suffer significant losses in efficiency compared with the maximum likelihood estimator. Therefore, a trade-off robustness-efficiency should be carefully analyzed.

Our contribution to robust portfolio optimization is developed within a minimum pseudodistance framework. We can say that the minimum pseudodistance methods for estimation take part to the same cathegory with the minimum divergence methods. The minimum divergence estimators are defined by minimizing some appropriate divergence between an assumed model and the true model underlying the data. Depending on the choice of the divergence, minimum divergence estimators can afford considerable robustness at minimal loss of efficiency. However, the classical approaches based on divergence minimization require nonparametric density estimation, which can be problematic in multi-dimensional settings. Some proposals to avoid the nonparametric density estimation in minimum divergence estimation have been made in [9, 10] and [11] and robustness properties of such estimators have been studied in [12], [13].

The pseudodistance that we use was originally introduced in [10], where it was called “type-0” divergence. Corresponding minimum divergence estimators have also been studied in [10]. It was also derived (using a cross entropy argument) and extensively studied in [14] where is called *γ*-divergence. More details about this divergence were provided in [15]. It was also introduced in [16] in the context of decomposable pseudodistances. The minimum pseudodistance estimators for general parametric models have been presented in [16, 17] and consist in minimization of an empirical version of a pseudodistance between the assumed model and the true model underlying the data. These estimators have the advantages of not requiring any prior smoothing and conciliate robustness with high efficiency, usually requiring distinct techniques. In some papers, the name of pseudodistance was used instead of divergence since it was considered that the divergences satisfy the information processing property, which is not the case of the pseudodistance. The interest on statistical methods based on information measures and particularly on divergences has grown substantially in recent years. We refer to the monographs [15, 18] for description of research and applications in this field, as well as to some recent articles [8, 19–21] developing such methods in applicative directions.

The contribution of the present paper is as follows. First we prove and discuss theoretical properties of the minimum pseudodistance estimators of multivariate location and covariance in the case of multivariate normal distribution, such as affine equivariance, B-robustness, asymptotic normality and asymptotic relative efficiency, as well as empirical properties based on Monte Carlo simulations. The behavior of the estimators depends on a tuning positive parameter *α* which controls the trade-off between robustness and efficiency. When the data are consistent with normality and *α* → 0, the estimation method corresponds to the maximum likelihood method (MLE) which is known to have full asymptotic efficiency at the model. When *α* > 0, the estimator gains robustness, while keeping high efficiency. The minimum pseudodistance estimators can be easily used in place of the classical mean and covariance matrix estimators, thereby providing robust and efficient mean-variance optimized portfolios. We prove asymptotic properties of portfolio weights based on minimum pseudodistance estimators, such as B-robustness and asymptotic normality. Then, on the basis of real data, we analyze different robust portfolios based on robust minimum pseudodistance estimators, studying both their in-sample and out-of-sample behavior and comparing them with some other portfolios known in literature. Among the analyzed portfolios count: optimal mean-variance portfolios using the classical MLE or minimum pseudodistance estimators or S-estimators, minimum-variance portfolios using the classical MLE or minimum pseudodistance estimators or S-estimators, as well as the equally-weighted portfolio. We considered the cases when short selling is allowed, respectively when short selling is not allowed. The out-of-sample empirical performance of the considered portfolios is evaluated using the measures: turnover, the out-of-sample portfolio variance and the out-of-sample Sharpe ratio. Our theoretical and numerical results show that the optimal portfolios based on minimum pseudodistance estimators are much more stable to extreme events than those obtained by plugging-in the MLEs and compare well with other optimal robust portfolios known in literature.

The outline of the paper is as follows: in Section 2, we shortly describe the Markowitz’s mean-variance model whose inputs are estimations of location and covariance of asset returns. The minimum pseudodistance estimators of location and covariance are introduced in Section 3. Here we prove theoretical properties of these estimators in the case of multivariate normal distribution, such as the affine equivariance and B-robustness. We also determine the asymptotic covariance matrices of the estimators and discuss the asymptotic relative efficiency. The estimators of the portfolio weights together with their asymptotic properties are presented in Section 4. In Sections 5 and 6, a Monte Carlo simulation study and then applications on real data show the advantages of the new approach. Here we illustrate both in-sample as well out-of-sample performance of the portfolios.

## Portfolio optimization model

We consider a portfolio formed by *N* financial assets. The returns of the assets are characterized by the random vector *X* = (*X*_1_, …, *X*_*N*_)^⊤^, where *X*_*i*_ denotes the random variable associated to the return of the asset *i*, *i* = 1, …, *N*. Let *p* = (*p*_1_, …, *p*_*N*_)^⊤^ be the vector of weights associated to the portfolio, where *p*_*i*_ represents the proportion of the investor’s capital invested in the asset *i*. The total return of the portfolio is given by the random variable

$$\begin{array}{c} p^{⊤}X=p_{1}X_{1}+\cdots +p_{N}X_{N}. \end{array}$$

Supposing that the random vector *X* follows a multivariate normal distribution $N_{N}(\mu ,\Sigma )$, where *μ* is the vector containing the mean returns of the assets and Σ is the covariance matrix of the returns of the assets, the mean of the portfolio return can be written as *R*(*p*) = *p* ^⊤^ *μ* and the portfolio variance as *S*(*p*) = *p* ^⊤^Σ*p*.

The Markowitz approach for optimal portfolio selection (see [1]) consists in solving the following optimization problem. For a given investor’s risk aversion *λ* > 0, the mean-variance optimization selects the optimal portfolio p*, solution of

$$\begin{array}{c} \arg\;\max_{p}\left\{R\left(p\right)-\frac{\lambda }{2}S\left(p\right)\right\} \end{array}$$ (1)

with the constraint *p* ^⊤^ *e*_*N*_ = 1, *e*_*N*_ being the *N* × 1 vector of ones. The set of optimal portfolios for all possible values of the risk aversion parameter *λ* defines the mean-variance efficient frontier. The solution of the above optimization problem is explicit and the optimal portfolio weights, for a fixed value of *λ*, are given by

$$\begin{array}{c} p^{*}=\frac{1}{\lambda }\Sigma^{-1}\left(\mu -\eta e_{N}\right) \end{array}$$ (2)

where

$$\begin{array}{c} \eta =\frac{e_{N}^{⊤}\Sigma^{-1}\mu -\lambda }{e_{N}^{⊤}\Sigma^{-1}e_{N}}. \end{array}$$

This is the case when short selling is allowed. When short selling is not allowed, we have a supplementary constraint in the optimization problem, namely all the weights *p*_*i*_ are positive.

When the true parameters *μ* and Σ and the portfolio weights are all known, then we have the true efficient frontier. An estimated efficient frontier can be obtained by using estimators of the mean and covariance matrix. Throughout this paper we denote by $\hat{\mu }$ and $\hat{\Sigma }$ the estimators of the parameters *μ* and Σ, and by $\hat{p^{*}}$ the estimator of the optimal portfolio weights, as resulting with Eq (2)

$$\begin{array}{c} \hat{p^{*}}=\frac{1}{\lambda }{\hat{\Sigma }}^{-1}[\hat{\mu }-\frac{e_{N}^{⊤}{\hat{\Sigma }}^{-1}\hat{\mu }-\lambda }{e_{N}^{⊤}{\hat{\Sigma }}^{-1}e_{N}}e_{N}]. \end{array}$$ (3)

The mean and the covariance matrix of the returns are in practice estimated by their sample counterparts, i.e. the maximum likelihood estimators under the multivariate normal model. It is known that, under normality, the maximum likelihood estimators are the most efficient. However, in the presence of outlying observations, the asymptotic bias of these estimators can be arbitrarily large and this bias is induced to the corresponding optimal portfolio weights. For this reason, *μ* and Σ should be robustly estimated.

## Robust estimators of the location and covariance

### Minimum pseudodistance estimators

For two probability measures *P* and *Q*, admitting densities *p*, respectively *q* with respect to the Lebesgue measure, the pseudodistances that we consider are defined through

$$\begin{array}{c} R_{\alpha }\left(P,Q\right):=\frac{1}{\alpha +1}\ln\int p^{\alpha }dP+\frac{1}{\alpha (\alpha +1)}\ln\int q^{\alpha }dQ-\frac{1}{\alpha }\ln\int p^{\alpha }dQ \end{array}$$ (4)

for *α* > 0 and satisfy the limit relation

$$\begin{array}{c} R_{\alpha }\left(P,Q\right)\to R_{0}\left(P,Q\right):=\int \ln\frac{q}{p}dQ\;\;\text{for}\;\;\alpha ↓0. \end{array}$$

Note that *R*_0_(*P*, *Q*) coincides with the modified Kullback-Leibler divergence. More details about Eq (4) and corresponding minimum divergence estimators can be found in [10, 14–16].

Let P be a parametric model with parameter space $\Theta \subset {\mathbb{R}}^{d}$ and assume that every probability measure *P*_*θ*_ in P has a density *p*_*θ*_ with respect to the Lebesgue measure. The family of minimum pseudodistance estimators of the unknown parameter *θ*_0_ is obtained by replacing the hypothetical probability measure *P*_*θ*_0__ in the pseudodistances *R*_*α*_(*P*_*θ*_, *P*_*θ*_0__) by the empirical measure *P*_*n*_ pertaining to the sample and then minimizing *R*_*α*_(*P*_*θ*_, *P*_*n*_) with respect to *θ* on the parameter space. We note that the pseudodistances *R*_*α*_ are also used in [22] in order to define optimal robust M-estimators using the Hampel infinitesimal approach.

Let *X* ^1^, …, *X* ^*T*^ be a sample on $X∼N_{N}(\mu ,\Sigma )$ and denote by *θ* = (*μ*, Σ) the parameter of interest. A minimum pseudodistance estimator $\hat{\theta }=(\hat{\mu },\hat{\Sigma })$ of *θ* is defined by

$$\begin{array}{c} \hat{\theta }:=\arg\underset{\theta }{\inf}R_{\alpha }\left(P_{\theta },P_{n}\right) \end{array}$$

which can be written equivalently as

$$\begin{array}{c} \hat{\theta }=\{\begin{array}{cc} \arg\underset{\theta }{\sup}\frac{1}{TC_{\alpha }\left(\theta \right)}\sum_{i=1}^{T}p_{\theta }^{\alpha }\left(X^{i}\right) & \;\;\;\;\text{if}\;\alpha >0\\ \arg\underset{\theta }{\sup}\frac{1}{T}\sum_{i=1}^{T}\ln p_{\theta }\left(X^{i}\right) & \;\;\;\;\text{if}\;\alpha =0 \end{array} \end{array}$$ (5)

where *p*_*θ*_ is the *N*-variate normal density and $C_{\alpha }(\theta )={\left(\int p_{\theta }^{\alpha +1}d\lambda \right)}^{\frac{\alpha }{\alpha +1}}$. Note that, the choice *α* = 0 leads to the definition of the classical MLE. Throughout the paper we will also use the notation ‖*x* − *μ*‖_Σ^−1^_: = [(*x* − *μ*)^⊤^Σ^−1^(*x* − *μ*)]^1/2^. A simple calculation shows that

$$\begin{array}{c} C_{\alpha }\left(\theta \right)=\frac{{\left(\frac{1}{2\pi }\right)}^{\frac{N\alpha^{2}}{2(\alpha +1)}}{\left(\sqrt{\det\Sigma^{-1}}\right)}^{\frac{\alpha^{2}}{\alpha +1}}}{{\left(\sqrt{\alpha +1}\right)}^{\frac{N\alpha }{\alpha +1}}}. \end{array}$$

For *α* > 0, the estimator Eq (5) can be expressed as

$$\begin{array}{c} \hat{\theta }=\arg\underset{\theta }{\sup}{\left(\sqrt{\det\Sigma^{-1}}\right)}^{\frac{\alpha }{\alpha +1}}\sum_{i=1}^{T}\exp(-\frac{\alpha }{2}{∥X^{i}-\mu ∥}_{\Sigma^{-1}}^{2}). \end{array}$$

By direct differentiation with respect to *μ* and Σ, we see that the estimators of these parameters are solutions of the system

$$\begin{array}{ccc} & & \sum_{i=1}^{T}\left(X^{i}-\mu \right)\exp(-\frac{\alpha }{2}{∥X^{i}-\mu ∥}_{\Sigma^{-1}}^{2})=0\\ & & \;\sum_{i=1}^{T}[\frac{1}{\alpha +1}\Sigma -{\left(X^{i}-\mu \right)\left(X^{i}-\mu \right)}^{t}]\exp(-\frac{\alpha }{2}{∥X^{i}-\mu ∥}_{\Sigma^{-1}}^{2})=0 \end{array}$$

which can be rewritten as

$$\begin{array}{ccc} \mu & = & \sum_{i=1}^{T}\frac{\exp(-\frac{\alpha }{2}{∥X^{i}-\mu ∥}_{\Sigma^{-1}}^{2})}{\sum_{i=1}^{T}\exp\left(-\frac{\alpha }{2}{∥X^{i}-\mu ∥}_{\Sigma^{-1}}^{2}\right)}X^{i} \end{array}$$ (6)

$$\begin{array}{ccc} \Sigma & = & \sum_{i=1}^{T}\frac{\left(\alpha +1\right)\exp(-\frac{\alpha }{2}{∥X^{i}-\mu ∥}_{\Sigma^{-1}}^{2})}{\sum_{i=1}^{T}\exp\left(-\frac{\alpha }{2}{∥X^{i}-\mu ∥}_{\Sigma^{-1}}^{2}\right)}\left(X^{i}-\mu \right){\left(X^{i}-\mu \right)}^{⊤}. \end{array}$$ (7)

### Affine equivariance

The location and dispersion estimators defined above are affine equivariant. More precisely, if $\hat{\mu }(\text{X})$ and $\hat{\Sigma }(\text{X})$ are estimators corresponding to a sample **X** = (*X* ^1^, …, *X* ^*T*^), then

$$\begin{array}{ccc} \hat{\mu }\left(AX+b\right) & = & A\hat{\mu }\left(X\right)+b \end{array}$$ (8)

$$\begin{array}{ccc} \hat{\Sigma }\left(AX+b\right) & = & A\hat{\Sigma }\left(X\right)A^{⊤} \end{array}$$ (9)

for any *N* × *N* nonsingular matrix *A* and any $b\in {\mathbb{R}}^{N}$. Indeed, let *A* be a nonsingular matrix, $b\in {\mathbb{R}}^{N}$ and **Y** = (*Y* ^1^, …, *Y* ^*T*^), *Y* ^*i*^: = *AX* ^*i*^ + *b*. Estimators $\hat{\mu }(\text{Y})$ and $\hat{\Sigma }(\text{Y})$ are solutions of the system obtained from Eqs (6) and (7) by replacing *X* ^*i*^ with *Y* ^*i*^. Then, by replacing *Y* ^*i*^ with *AX* ^*i*^+*b*, we find $\hat{\mu }(\text{X})=A^{-1}(\hat{\mu }(\text{Y})-b)$ and $\hat{\Sigma }(\text{X})=A^{-1}\hat{\Sigma }(\text{Y}){\left(A^{⊤}\right)}^{-1}$. Hence, Eqs (8) and (9) hold.

### Influence functions

A fundamental tool used for studying statistical robustness is the influence function. Recall that, a map *T* defined on a set of probability measures and parameter space valued is a statistical functional corresponding to an estimator ${\hat{\theta }}_{n}$ of the parameter *θ*, if ${\hat{\theta }}_{n}=T(P_{n})$, where *P*_*n*_ is the empirical measure associated to the sample. The influence function of *T* at *P*_*θ*_ is defined by

$$\begin{array}{c} \text{IF}\left(x;T,P_{\theta }\right):={\frac{\partial T({\tilde{P}}_{\varepsilon x})}{\partial \varepsilon }|}_{\varepsilon =0} \end{array}$$

where ${\tilde{P}}_{ɛx}:=(1-ɛ)P_{\theta }+ɛ\delta_{x},$ *ɛ* > 0, *δ*_*x*_ being the Dirac measure putting all mass at *x*. Whenever the influence function is bounded with respect to *x* the corresponding estimator is called robust (see [23]).

The statistical functionals associated to the minimum pseudodistance estimators of *μ* and Σ are *μ*(*P*) and Σ(*P*) defined by the solutions of the system

$$\begin{array}{ccc} & & \int \left(x-\mu \right)\exp(-\frac{\alpha }{2}{∥x-\mu ∥}_{\Sigma^{-1}}^{2})dP\left(x\right)=0\\ & & \int \;[\left(x-\mu \right){\left(x-\mu \right)}^{⊤}\exp(-\frac{\alpha }{2}{∥x-\mu ∥}_{\Sigma^{-1}}^{2})-\\ & & \;\;\;-\frac{1}{\alpha +1}\Sigma \exp(-\frac{\alpha }{2}{∥x-\mu ∥}_{\Sigma^{-1}}^{2})]dP\left(x\right)=0. \end{array}$$

This system can be rewritten under the form

$$\begin{array}{c} \int w_{1}\left({∥x-\mu ∥}_{\Sigma^{-1}}\right)\left(x-\mu \right)dP\left(x\right)=0 \end{array}$$ (10)

$$\begin{array}{c} \int [\frac{w_{2}\left({∥x-\mu ∥}_{\Sigma^{-1}}\right)}{{∥x-\mu ∥}_{\Sigma^{-1}}^{2}}\left(x-\mu \right){\left(x-\mu \right)}^{⊤}-w_{3}\left({∥x-\mu ∥}_{\Sigma^{-1}}\right)\Sigma ]dP\left(x\right)=0 \end{array}$$ (11)

where

$$\begin{array}{c} w_{1}\left(t\right)=\exp(-\frac{\alpha }{2}t^{2})\;,w_{2}\left(t\right)=\exp(-\frac{\alpha }{2}t^{2})t^{2},\;w_{3}\left(t\right)=\frac{1}{\alpha +1}\exp(-\frac{\alpha }{2}t^{2}). \end{array}$$ (12)

We note that the solutions of the system given by Eqs (10) and (11), when *w*_1_, *w*_2_, *w*_3_ are arbitrary weight functions, define statistical functionals of general M-estimators of (*μ*, Σ) (see [24] and [25]). According to the results presented in [25], the influence functions for general M-estimators of *μ* and Σ are given by

$$\begin{array}{ccc} \text{IF}(x;\mu ,P_{\mu ,\Sigma }) & = & \left(x-\mu \right)w_{\mu }\left({∥x-\mu ∥}_{\Sigma^{-1}}\right) \end{array}$$ (13)

$$\begin{array}{ccc} \text{IF}(x;\Sigma ,P_{\mu ,\Sigma }) & = & \left(x-\mu \right){\left(x-\mu \right)}^{⊤}w_{\eta }\left({∥x-\mu ∥}_{\Sigma^{-1}}\right)-\Sigma w_{\delta }\left({∥x-\mu ∥}_{\Sigma^{-1}}\right) \end{array}$$ (14)

where

$$\begin{array}{ccc} w_{\mu }\left({∥x-\mu ∥}_{\Sigma^{-1}}\right) & = & \frac{w_{1}\left({∥x-\mu ∥}_{\Sigma^{-1}}\right)}{E_{P_{0}}[w_{1}\left(∥y∥\right)+\frac{1}{N}w_{1}^{\prime }(∥y∥)∥y∥]}\\ w_{\eta }\left({∥x-\mu ∥}_{\Sigma^{-1}}\right) & = & \frac{N\left(N+2\right)w_{2}\left({∥x-\mu ∥}_{\Sigma^{-1}}\right)}{{∥x-\mu ∥}_{\Sigma^{-1}}^{2}E_{P_{0}}[Nw_{2}\left(∥y∥\right)+w_{2}^{\prime }(∥y∥)∥y∥]}\\ w_{\delta }\left({∥x-\mu ∥}_{\Sigma^{-1}}\right) & = & \frac{Nw_{3}\left({∥x-\mu ∥}_{\Sigma^{-1}}\right)-2w_{2}\left({∥x-\mu ∥}_{\Sigma^{-1}}\right)}{E_{P_{0}}[w_{2}^{\prime }(∥y∥)∥y∥-Nw_{3}^{\prime }(∥y∥)∥y∥]}+\\ & & +\frac{\left(N+2\right)w_{2}\left({∥x-\mu ∥}_{\Sigma^{-1}}\right)}{E_{P_{0}}[Nw_{2}\left(∥y∥\right)+w_{2}^{\prime }(∥y∥)∥y∥]} \end{array}$$

*P*_0_ denoting the probability measure associate to the *N*-variate standard normal distribution and ‖⋅‖ the Euclidian norm.

For the weight functions *w*_1_, *w*_2_, *w*_3_ from Eq (12), corresponding to the minimum pseudodistance estimators, we get

$$\begin{array}{ccc} w_{\mu }\left(t\right) & = & {\left(\sqrt{\alpha +1}\right)}^{N+2}\exp(-\frac{\alpha }{2}t^{2}) \end{array}$$ (15)

$$\begin{array}{ccc} w_{\eta }\left(t\right) & = & {\left(\sqrt{\alpha +1}\right)}^{N+4}\exp(-\frac{\alpha }{2}t^{2}) \end{array}$$ (16)

$$\begin{array}{ccc} w_{\delta }\left(t\right) & = & {\left(\sqrt{\alpha +1}\right)}^{N+2}\exp(-\frac{\alpha }{2}t^{2}) \end{array}$$ (17)

and replacing in Eqs (13) and (14) we obtain

$$\begin{array}{ccc} \text{IF}(x;\mu ,P_{\mu ,\Sigma }) & = & {\left(\sqrt{\alpha +1}\right)}^{N+2}\left(x-\mu \right)\exp(-\frac{\alpha }{2}{∥x-\mu ∥}_{\Sigma^{-1}}^{2}) \end{array}$$ (18)

$$\begin{array}{ccc} \text{IF}(x;\Sigma ,P_{\mu ,\Sigma }) & = & \;{\left(\sqrt{\alpha +1}\right)}^{N+4}\;[\left(x-\mu \right){\left(x-\mu \right)}^{⊤}\;\;-\;\frac{1}{\alpha +1}\Sigma ]\exp(\;-\frac{\alpha }{2}{∥x-\mu ∥}_{\Sigma^{-1}}^{2}). \end{array}$$ (19)

Both influence functions are bounded with respect to *x*. Therefore the minimum pseudodistance estimators of *μ* and Σ are robust.

**Fig 1:**
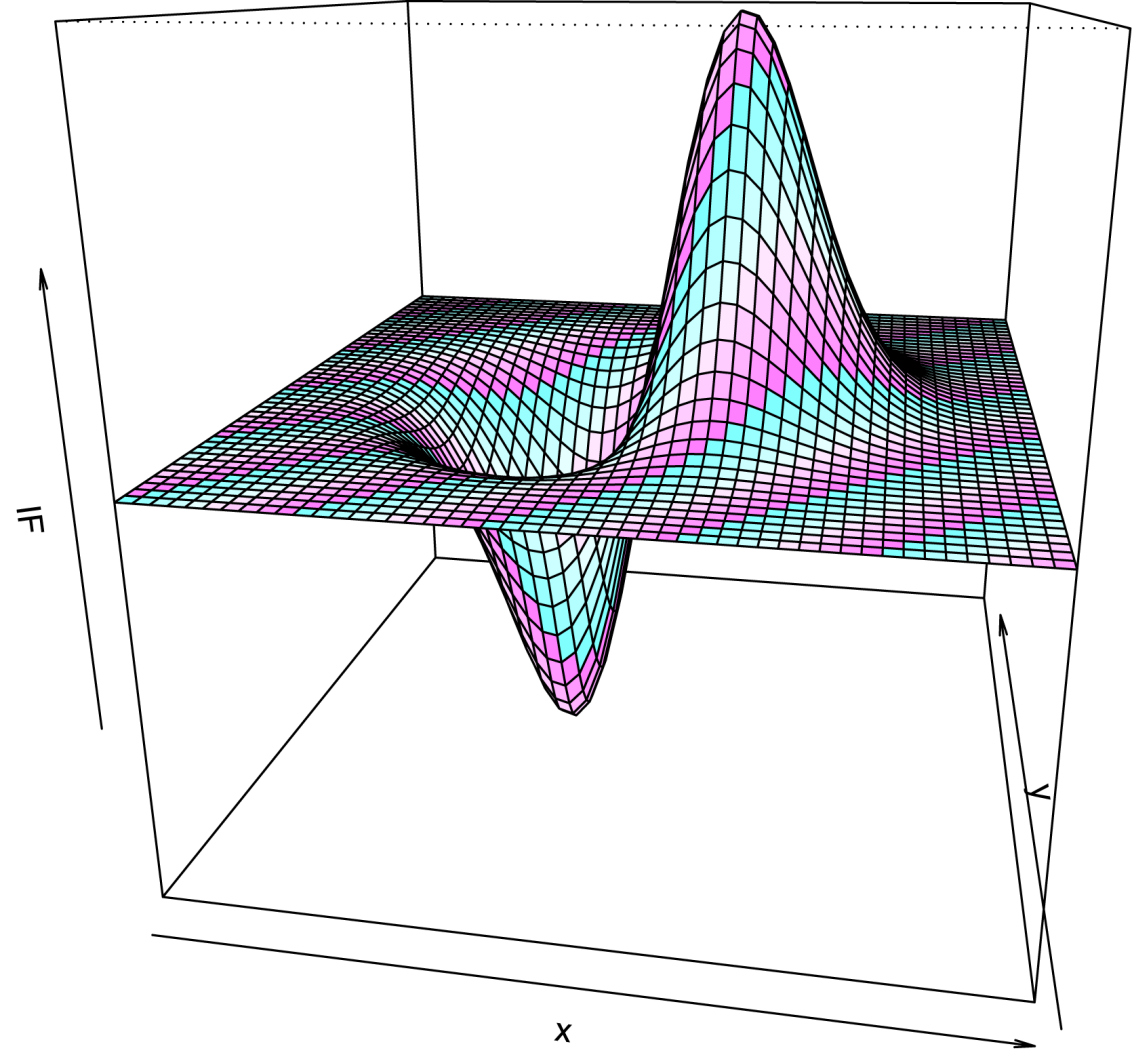
The influence function for the first component of the minimum pseudodistance estimator of the mean.

In Fig 1 the influence function for the first component of the minimum pseudodistance estimator of the mean is represented. Here *P*_0_ is the bivariate standard normal law and the constant corresponding to the estimator was chosed *α* = 0.5. The influence function is bounded and also redescending. A major feature of the minimum pseudodistance estimators considered in this paper is that they are redescending (the influence functions tend to 0, when ∣∣*x*∣∣ tends to infinity) and this represents an important advantage from the robustness point of view.

### Asymptotic normality

For general parametric models, under some regularity conditions, the minimum pseudodistance estimators are asymptotically normal distributed (see [16]). In this section, we derive the asymptotic covariance matrices of the mean and the covariance matrix minimum pseudodistance estimators. We adopt the influence function approach and make use of the general results for affine equivariant location and dispersion M-estimators as presented in [26] and [23].

When the observations correspond to the standard *N*-variate normal law *P*_0_, under appropriate conditions, $\hat{\mu }$ is asymptotically normal distributed with the asymptotic covariance matrix

$$\begin{array}{c} V\left(\mu ,P_{0}\right)=E_{P_{0}}\left\{\text{IF}\left(Z;\mu ,P_{0}\right)\text{IF}{\left(Z;\mu ,P_{0}\right)}^{⊤}\right\}=d_{\mu }I \end{array}$$ (20)

where $d_{\mu }:={\text{E}}_{P_{0}}\{{\left\|Z\right\|}^{2}w_{\mu }^{2}(\|Z\|)\}/N$ and *I* is the identity matrix. Formula (20) has been established in [26] for general affine equivariant location M-estimators. The estimator $\hat{\mu }$ belongs to this class. For the weight *w*_*μ*_ from Eq (15) we get $d_{\mu }={\left(\alpha +1\right)}^{N+2}/{\left(\sqrt{2\alpha +1}\right)}^{N+2}$, hence the asymptotic covariance matrix of the minimum pseudodistance estimator $\hat{\mu }$ is

$$\begin{array}{c} V\left(\mu ,P_{0}\right)={\left(\frac{\alpha +1}{\sqrt{2\alpha +1}}\right)}^{N+2}I. \end{array}$$

When the observations correspond to the normal law *P*_*μ*,Σ_, the asymptotic covariance matrix of $\hat{\mu }$ is given by

$$\begin{array}{ccc} V(\mu ,P_{\mu ,\Sigma }) & = & E_{P_{\mu ,\Sigma }}\left\{\text{IF}\left(X;\mu ,P_{\mu ,\Sigma }\right)\text{IF}{\left(X;\mu ,P_{\mu ,\Sigma }\right)}^{⊤}\right\}\\ & = & d_{\mu }\Sigma ={\left(\frac{\alpha +1}{\sqrt{2\alpha +1}}\right)}^{N+2}\Sigma . \end{array}$$ (21)

Similar results hold for $\text{vecs}(\hat{\Sigma })$, where vecs is the operation that stacks the *N* + *N*(*N* − 1)/2 non-redundant elements of Σ into a vector, as follows:

$$\begin{array}{c} \text{vecs}\left(\Sigma \right):={\left(\sigma_{11}/\sqrt{2},\cdots ,\sigma_{NN}/\sqrt{2},\sigma_{21},\sigma_{31},\cdots ,\sigma_{N,N-1}\right)}^{⊤}. \end{array}$$

According to the results presented in [26], when the observations come from the *N*-variate standard normal law *P*_0_, the asymptotic covariance matrix corresponding to an affine equivariant M-estimator of the covariance matrix is given by

$$\begin{array}{ccc} V(\Sigma ,P_{0}) & = & E_{P_{0}}\left\{\text{vecsIF}\left(Z;\Sigma ,P_{0}\right)\text{vecsIF}{\left(Z;\Sigma ,P_{0}\right)}^{⊤}\right\}\\ & = & d_{\eta }\left(I-\frac{1}{N}ww^{⊤}\right)+d_{\tau }\cdot \frac{1}{N}ww^{⊤} \end{array}$$

where $w^{⊤}:=(e_{N}^{⊤},{\text{0}}_{N(N-1)/2}^{⊤})$, $d_{\eta }:={\text{E}}_{P_{0}}\{{\left\|Z\right\|}^{4}w_{\eta }^{2}(\|Z\|)\}/(N(N+2))$ and $d_{\tau }:={\text{E}}_{P_{0}}\{w_{\tau }^{2}(\|Z\|)\}/(2N)$ with *w*_*τ*_: = *t* ^2^ *w*_*η*_(*t*) − *Nw*_*δ*_(*t*), *w*_*η*_, *w*_*δ*_ and *w*_*τ*_ being specific to the M-estimator in question.

In our case, *w*_*η*_ and *w*_*δ*_ are given by Eqs (16) and (17), hence

$$\begin{array}{c} w_{\tau }\left(t\right)={\left(\sqrt{\alpha +1}\right)}^{N+4}[t^{2}-\frac{N}{\alpha +1}]\exp(-\frac{\alpha }{2}t^{2}). \end{array}$$

After some calculation, we obtain

$$\begin{array}{c} d_{\eta }={\left(\frac{\alpha +1}{\sqrt{2\alpha +1}}\right)}^{N+4}\;\;\text{and}\;\;d_{\tau }=\frac{N\alpha^{2}{\left(\alpha +1\right)}^{N+2}}{2{\left(\sqrt{2\alpha +1}\right)}^{N+4}}+{\left(\frac{\alpha +1}{\sqrt{2\alpha +1}}\right)}^{N+4}, \end{array}$$

therefore,

$$\begin{array}{c} V\left(\Sigma ,P_{0}\right)={\left(\frac{\alpha +1}{\sqrt{2\alpha +1}}\right)}^{N+4}I+\frac{\alpha^{2}{\left(\alpha +1\right)}^{N+2}}{2{\left(\sqrt{2\alpha +1}\right)}^{N+4}}ww^{⊤}. \end{array}$$ (22)

When the observations correspond to the law *P*_*μ*,Σ_, the asymptotic covariance matrix of $\text{vecs}(\hat{\Sigma })$ can be established by using the formula from [23] p.282, which in our notations writes as follows

$$\begin{array}{c} V\left(\Sigma ,P_{\mu ,\Sigma }\right)=[\frac{\partial \text{vecs}[\Sigma^{\frac{1}{2}}S\Sigma^{\frac{1}{2}}]}{\partial \text{vecs}S}]V\left(\Sigma ,P_{0}\right){\left[\frac{\partial \text{vecs}[\Sigma^{\frac{1}{2}}S\Sigma^{\frac{1}{2}}]}{\partial \text{vecs}\Sigma }\right]}^{⊤}. \end{array}$$ (23)

According to [23] p.272, for a given *N* × *N* matrix Σ_*_, it holds

$$\begin{array}{c} {}[\frac{\partial \text{vecs}[\Sigma^{\frac{1}{2}}S\Sigma^{\frac{1}{2}}]}{\partial \text{vecs}S}]\text{vecs}\Sigma_{*}=\text{vecs}\left(\Sigma^{\frac{1}{2}}\Sigma_{*}\Sigma^{\frac{1}{2}}\right). \end{array}$$

Particularly,

$$\begin{array}{c} {}[\frac{\partial \text{vecs}[\Sigma^{\frac{1}{2}}S\Sigma^{\frac{1}{2}}]}{\partial \text{vecs}S}]\text{vecs}I=\text{vecs}\Sigma . \end{array}$$ (24)

Note that $w=\sqrt{2}\text{vecs}I$ and combining Eqs (23), (22) and (24), we get

$$\begin{array}{ccc} V(\Sigma ,P_{\mu ,\Sigma }) & = & {\left(\frac{\alpha +1}{\sqrt{2\alpha +1}}\right)}^{N+4}[\frac{\partial \text{vecs}[\Sigma^{\frac{1}{2}}S\Sigma^{\frac{1}{2}}]}{\partial \text{vecs}S}]{\left[\frac{\partial \text{vecs}[\Sigma^{\frac{1}{2}}S\Sigma^{\frac{1}{2}}]}{\partial \text{vecs}S}\right]}^{⊤}+\\ & & +\;\frac{\alpha^{2}{\left(\alpha +1\right)}^{N+2}}{{\left(\sqrt{2\alpha +1}\right)}^{N+4}}\text{vecs}\Sigma {\left(\text{vecs}\Sigma \right)}^{⊤}. \end{array}$$

For symmetry reasons, the minimum pseudodistance location and covariance estimators are asymptotically uncorrelated and hence asymptotically independent. This is valid for location and covariance M-estimators in general, as it is underlined in various articles, for example in [24].

### Asymptotic relative efficiency

In order to assess the efficiency of the proposed estimators with respect to that of the MLE, we adopt as measure the asymptotic relative efficiency (ARE). For a parameter *θ* taking values in ${\mathbb{R}}^{d}$ and an estimator $\hat{\theta }$ which is asymptotically *d*-variate normal with mean *θ* and nonsingular covariance matrix *V*(*θ*, *P*), the asymptotic relative efficiency with respect to that of the MLE is defined as

$$\begin{array}{c} \text{ARE}\left(\hat{\theta },P\right)={\left(\frac{\det V_{0}\left(\theta ,P\right)}{\det V(\theta ,P)}\right)}^{1/d}, \end{array}$$

*V*_0_(*θ*, *P*) being the asymptotic covariance matrix of the MLE of *θ* when the observations follow the law *P* (see [27]). Although the asymptotically most efficient estimator is given by the MLE, the particular MLE can be drastically inefficient when the underlying distribution departs even a little bit from the assumed nominal distribution. Therefore the trade-off between robustness and efficiency should be carefully analyzed.

Due to the asymptotic independence of the mean and the covariance matrix minimum pseudodistance estimators, the asymptotic relative efficiency of $\hat{\theta }={\left({\hat{\mu }}^{⊤},\text{vecs}{\left(\hat{\Sigma }\right)}^{⊤}\right)}^{⊤}$ can be expressed as

$$\begin{array}{ccc} \text{ARE}(\hat{\theta },P_{\mu ,\Sigma }) & = & {\left(\frac{\det V_{0}\left(\theta ,P_{\mu ,\Sigma }\right)}{\det V(\theta ,P_{\mu ,\Sigma })}\right)}^{\frac{2}{N(N+3)}}\\ & = & {\left(\frac{\det V_{0}\left(\mu ,P_{\mu ,\Sigma }\right)\det V_{0}\left(\Sigma ,P_{\mu ,\Sigma }\right)}{\det V\left(\mu ,P_{\mu ,\Sigma }\right)\det V\left(\Sigma ,P_{\mu ,\Sigma }\right)}\right)}^{\frac{2}{N(N+3)}}. \end{array}$$ (25)

Using Eqs (21) and (23), formula (25) can be written as

$$\begin{array}{c} \text{ARE}\left(\hat{\theta },P_{\mu ,\Sigma }\right)={\left(\frac{\det V_{0}\left(\mu ,P_{0}\right)\det V_{0}\left(\Sigma ,P_{0}\right)}{\det V\left(\mu ,P_{0}\right)\det V\left(\Sigma ,P_{0}\right)}\right)}^{\frac{2}{N(N+3)}}. \end{array}$$

A direct calculation shows that

$$\begin{array}{ccc} \det V(\mu ,P_{0}) & = & {\left(\frac{\alpha +1}{\sqrt{2\alpha +1}}\right)}^{N(N+2)}\\ \det V(\Sigma ,P_{0}) & = & {\left(\frac{\alpha +1}{\sqrt{2\alpha +1}}\right)}^{\frac{N(N+1)(N+4)}{2}}(1+\frac{N\alpha^{2}}{2{\left(\alpha +1\right)}^{2}}). \end{array}$$

Particularly, for *α* = 0, we find the similar quantities for the MLE, namely det *V*_0_(*μ*, *P*_0_) = 1 and det *V*_0_(Σ, *P*_0_) = 1. Hence

$$\begin{array}{c} \text{ARE}\left(\hat{\theta },P_{\mu ,\Sigma }\right)=\frac{1}{{\left(\frac{\alpha +1}{\sqrt{2\alpha +1}}\right)}^{\frac{N^{2}+7N+8}{N+3}}{\left(1+\frac{N\alpha^{2}}{2{\left(\alpha +1\right)}^{2}}\right)}^{\frac{2}{N(N+3)}}}. \end{array}$$ (26)

Note that, for fixed *N* and *α*, $\text{ARE}(\hat{\theta },P_{\mu ,\Sigma })$ is the same, whatever *μ* or Σ.

In Table 1 values of the asymptotic relative efficiency Eq (26) are given. As it can be seen, when *N* or *α* increases, the asymptotic relative efficiency $\text{ARE}(\hat{\theta },P_{\mu ,\Sigma })$ decreases. Therefore, values of *α* close to zero will provide high efficiency and in the meantime the robustness of the estimation procedure.

**Table 1:** Asymptotic relative efficiency of the minimum pseudodistance estimators.

N	*α* = 0	*α* = 0.1	*α* = 0.2	*α* = 0.5	*α* = 0.75	*α* = 1
1	1	0.98151	0.93871	0.76904	0.63774	0.53033
2	1	0.97704	0.92429	0.72086	0.57042	0.45266
3	1	0.97273	0.91051	0.67698	0.51187	0.38814
4	1	0.96851	0.89718	0.63647	0.46018	0.33370
5	1	0.96435	0.88419	0.59879	0.41420	0.28738
6	1	0.96025	0.87148	0.56360	0.37311	0.24778
7	1	0.95619	0.85902	0.53065	0.33629	0.21380
8	1	0.95215	0.84679	0.49975	0.30322	0.18460
9	1	0.94815	0.83477	0.47073	0.27350	0.15946
10	1	0.94418	0.82294	0.44345	0.24674	0.13779

## The estimator of the optimal portfolio weights

We consider the estimator $\hat{p^{*}}$ of the optimal portfolio weights, as given by Eq (3), with $\hat{\mu }$ and $\hat{\Sigma }$ minimum pseudodistance estimators.

The influence function of the estimator $\hat{p^{*}}$ is proportional to the influence functions of the estimators $\hat{\mu }$ and $\hat{\Sigma }$. More precisely,

$$\begin{array}{ccc} \text{IF}(x;p^{*},P_{\mu ,\Sigma }) & = & -\Sigma^{-1}\text{IF}\left(x;\Sigma ,P_{\mu ,\Sigma }\right)p^{*}+\frac{1}{\lambda }\Sigma^{-1}\{\text{IF}(x;\mu ,P_{\mu ,\Sigma })+\\ & & +\frac{e_{N}^{⊤}\Sigma^{-1}\left[\text{IF}\left(x;\Sigma ,P_{\mu ,\Sigma }\right)\Sigma^{-1}\mu -\text{IF}\left(x;\mu ,P_{\mu ,\Sigma }\right)\right]e_{N}}{e_{N}^{⊤}\Sigma^{-1}e_{N}}+\\ & & +\frac{\left(e_{N}^{⊤}\Sigma^{-1}\text{IF}\left(x;\Sigma ,P_{\mu ,\Sigma }\right)\Sigma^{-1}e_{N}\right)\left(e_{N}^{⊤}\Sigma^{-1}\mu -\lambda \right)e_{N}}{{\left(e_{N}^{⊤}\Sigma^{-1}e_{N}\right)}^{2}}\} \end{array}$$ (27)

where IF(*x*; *μ*, *P*_*μ*,Σ_) and IF(*x*; Σ, *P*_*μ*,Σ_) are those from Eqs (18) and (19). This formula is obtained by considering the statistical functional associated to the optimal portfolio weights,

$$\begin{array}{c} p^{*}\left(P\right)=\frac{1}{\lambda }\Sigma^{-1}\left(P\right)[\mu \left(P\right)-\frac{e_{N}^{⊤}\Sigma^{-1}\left(P\right)\mu \left(P\right)-\lambda }{e_{N}^{⊤}\Sigma^{-1}\left(P\right)e_{N}}e_{N}] \end{array}$$

where Σ^−1^(*P*) denotes the statistical functional corresponding to ${\hat{\Sigma }}^{-1}$, and then deriving the influence function, taking also into account that

$$\begin{array}{c} \text{IF}\left(x;\Sigma^{-1},P_{\mu ,\Sigma }\right)=-\Sigma^{-1}\text{IF}\left(x;\Sigma ,P_{\mu ,\Sigma }\right)\Sigma^{-1}. \end{array}$$

On the basis of the direct proportionality between the influence function IF(*x*; p*, *P*_*μ*,Σ_) and the influence functions IF(*x*; *μ*, *P*_*μ*,Σ_) and IF(*x*; Σ, *P*_*μ*,Σ_), we deduce that the global robustness of $\hat{\mu }$ and $\hat{\Sigma }$ is transferred to the plug-in estimator $\hat{p^{*}}$.

The consistency of the estimator of the optimal portfolio weights can be obtained using continuity arguments (see also [28]).

Note that, for the market parameters (*μ*, Σ), Eq (1) is a convex optimization problem and possesses at least one optimal solution since the space of the feasible portfolios on which we optimize is compact. In addition, the solution is unique and is given by Eq (2). Since the problem (1) has a unique solution for each (*μ*, Σ) in a neighborhood of a given (*μ*_0_, Σ_0_), for a fixed *λ*, the mapping (*μ*, Σ) → p* = p*(*λ*, *μ*, Σ) is continuous in a neighborhood of (*μ*_0_, Σ_0_). Indeed, according to Theorem 4.2.1 from [29], the optimal set mapping is Hausdorff upper semicontinuous due to the compactness of the feasibility set and due to the continuity of the objective function. As the solution is unique, upper semicontinuity of the set-valued map yields continuity in the usual sense.

Then, for a given *λ*, ${\hat{p}}^{*}={\hat{p}}^{*}(\lambda ,\hat{\mu },\hat{\Sigma })$ is a consistent estimator of the true optimal portfolio weights $p^{*}=p^{*}(\lambda ,\hat{\mu },\hat{\Sigma })$, if $\hat{\mu },\hat{\Sigma }$ are consistent estimators of *μ* and Σ. Indeed, the almost sure convergence and the convergence in probability remain valid after continuous transformations. The consistency of the estimators $\hat{\mu },\hat{\Sigma }$ is implied by arguments from the theory of M-estimators, as developed by Huber (see [24] p.176). Using the uniqueness of the optimal solution, we have the continuity of the function (*μ*, Σ) → p*(*λ*, *μ*, Σ), as noted above. Therefore, the consistency of the estimator of the optimal portfolio weights holds.

On the other hand, by using the multivariate Delta method, the asymptotic normality of $\hat{p^{*}}$ is kept, as well. Given the i.i.d. observations *X* ^1^, …, *X* ^*T*^ from *P*_*μ*,Σ_, since $\hat{\mu }$ and $\text{vecs}(\hat{\Sigma })$ are asymptotically normal and the function $h(\theta )=\frac{1}{\lambda }\Sigma^{-1}(\mu -\eta e_{N})$ with *θ* = (*μ* ^⊤^,(vecsΣ)^⊤^)^⊤^ is differentiable, by applying the multivariate Delta method, it holds $\sqrt{n}(\hat{p^{*}}-p^{*})\to N(0,V(p^{*},P_{\mu ,\Sigma }))$ where *V*(p*, *P*_*μ*,Σ_) = D*h*(*θ*)*V*(*θ*, *P*_*μ*,Σ_)D*h*(*θ*)^⊤^, D*h*(*θ*) is the differential of *h* in *θ* and

$$\begin{array}{c} V\left(\theta ,P_{\mu ,\Sigma }\right)=(\begin{array}{cc} V(\mu ,P_{\mu ,\Sigma }) & 0\\ 0 & V(\Sigma ,P_{\mu ,\Sigma }) \end{array}). \end{array}$$

## Monte Carlo simulations

We performed Monte Carlo simulations in order to assess the performance of the minimum pseudodistance estimators of the mean and covariance matrix, for both contaminated and non-contaminated data.

In a first study, we considered the multivariate normal distribution $N_{N}(\mu_{0},\Sigma_{0})$, with *μ*_0_ = **0** and Σ_0_ a *N* × *N* matrix with variances equal to 1 and covariances all equal to 0.2. We generated samples of size *T* in which about (1 − *ɛ*)*T* observations are from $N_{N}(\mu_{0},\Sigma_{0})$, while a smaller portion *ɛT* is from the contaminating distribution $N_{N}(\mu_{c},\Sigma_{c})$ with *μ*_*c*_ = **−4** and Σ_*c*_ = 4Σ_0_. We considered *N* ∈ {2, 5, 10, 20, 50} and *ɛ* ∈ {0, 0.05, 0.1, 0.2}. For each setting, we generated 1000 samples and for each sample we computed minimum pseudodistance estimates $\hat{\mu }$ and $\hat{\Sigma }$ corresponding to *α* ∈ {0, 0.1, 0.2, 0.5, 0.75, 1}.

The estimates $\hat{\mu }$ and $\hat{\Sigma }$, which are solutions of the system of Eqs (6) and (7), were obtained using the following reweighting algorithm.

Let *s* ∈ {0, 1, …, s*} denotes the iteration step.

1. If *s* = 0

*μ* ^(*s*)^ and Σ^(*s*)^ are set to be initial estimates of location and scale;

2. For 0 < *s* < s*,

$$\begin{array}{ccc} \mu^{\left(s\right)} & = & \sum_{i=1}^{T}w_{i}^{\left(s-1\right)}X^{i}\\ \Sigma^{\left(s\right)} & = & \sum_{i=1}^{T}\left(\alpha +1\right)w_{i}^{\left(s-1\right)}\left(X^{i}-\mu^{\left(s\right)}\right){\left(X^{i}-\mu^{\left(s\right)}\right)}^{⊤} \end{array}$$

where

$$\begin{array}{c} w_{i}^{\left(s\right)}=\frac{\exp(-\frac{\alpha }{2}{\left(X^{i}-\mu^{\left(s\right)}\right)}^{⊤}{\left(\Sigma^{\left(s\right)}\right)}^{-1}\left(X^{i}-\mu^{\left(s\right)}\right))}{\sum_{i=1}^{T}\exp(-\frac{\alpha }{2}{\left(X^{i}-\mu^{\left(s\right)}\right)}^{⊤}{\left(\Sigma^{\left(s\right)}\right)}^{-1}\left(X^{i}-\mu^{\left(s\right)}\right))}. \end{array}$$

At step 1, we used maximum likelihood estimates as initial estimates of location and covariance. For details on general convergence behavior of reweighting algorithms we refer to [30]. If *α* > 0, the above procedure associates low weights to the observations that disagree sensibly with the model. If *α* = 0, all the observations receive the same weight and the estimators are the maximum likelihood ones

$$\begin{array}{ccc} {\hat{\mu }}_{ML} & = & \frac{1}{T}\sum_{i=1}^{T}X^{i}\\ {\hat{\Sigma }}_{ML} & = & \frac{1}{T}\sum_{i=1}^{T}\left(X^{i}-{\hat{\mu }}_{ML}\right){\left(X^{i}-{\hat{\mu }}_{ML}\right)}^{⊤}. \end{array}$$

We present simulation based estimates of the mean square error given by

$$\begin{array}{c} \hat{\text{MSE}}=\frac{1}{n_{s}}\sum_{i=1}^{n_{s}}{∥{\hat{\theta }}_{i}-\theta_{0}∥}^{2} \end{array}$$

where *n*_*s*_ is the number of samples (in our case *n*_*s*_ = 1000), $\theta_{0}={\left(\mu_{0}^{⊤},\text{vech}{\left(\Sigma_{0}\right)}^{⊤}\right)}^{⊤}$ and ${\hat{\theta }}_{i}={\left({\hat{\mu }}_{i}^{⊤},\text{vech}{\left({\hat{\Sigma }}_{i}\right)}^{⊤}\right)}^{⊤}$ is an estimation corresponding to the sample *i*. Here vech(Σ) is “the vector half”, namely the *n*(*n*+1)/2-dimensional column vector obtained by stacking the columns of the lower triangle of Σ, including the diagonal, one below the other.

Tables 2 and 3 present simulation based estimates of the mean square error, when the sample size is *T* = 10 ⋅ *N*, respectively when *T* = 100 ⋅ *N*.

**Table 2:** Simulation based estimates of the mean square error, when *T* = 10 ⋅ *N*.

*ɛ* = 0%						
N	*α* = 0	*α* = 0.1	*α* = 0.2	*α* = 0.5	*α* = 0.75	*α* = 1
2	0.343	0.358	0.384	0.559	0.804	1.177
5	0.513	0.530	0.593	1.340	4.806	5.324
10	0.760	0.817	0.945	10.471	11.389	12.197
20	1.290	1.429	2.069	29.979	38.830	47.064
*ɛ* = 5%						
N	*α* = 0	*α* = 0.1	*α* = 0.2	*α* = 0.5	*α* = 0.75	*α* = 1
2	4.425	0.888	0.533	0.593	0.849	1.202
5	18.816	1.077	0.662	1.565	4.985	5.437
10	41.312	0.951	1.022	10.646	11.470	12.600
20	145.172	1.517	2.273	30.339	39.561	47.072
*ɛ* = 10%						
N	*α* = 0	*α* = 0.1	*α* = 0.2	*α* = 0.5	*α* = 0.75	*α* = 1
2	11.554	4.605	0.945	0.694	0.923	1.294
5	43.446	4.075	0.749	1.758	5.052	5.449
10	143.395	1.325	1.091	10.720	11.454	12.648
20	503.319	1.648	2.422	30.776	39.758	47.693
*ɛ* = 20%						
N	*α* = 0	*α* = 0.1	*α* = 0.2	*α* = 0.5	*α* = 0.75	*α* = 1
2	32.696	24.612	9.955	1.118	1.190	1.475
5	132.542	53.841	1.869	2.171	5.362	5.625
10	441.209	19.233	1.241	10.751	11.751	12.745
20	1613.373	1.930	3.742	31.361	40.292	49.644

**Table 3:** Simulation based estimates of the mean square error, when *T* = 100 ⋅ *N*.

*ɛ* = 0%						
N	*α* = 0	*α* = 0.1	*α* = 0.2	*α* = 0.5	*α* = 0.75	*α* = 1
2	0.035	0.035	0.039	0.051	0.067	0.084
5	0.050	0.052	0.057	0.087	0.135	0.204
10	0.075	0.081	0.093	0.185	0.395	8.703
20	0.129	0.142	0.181	0.910	28.127	31.790
50	0.287	0.359	0.685	171.329	219.263	262.867
*ɛ* = 5%						
N	*α* = 0	*α* = 0.1	*α* = 0.2	*α* = 0.5	*α* = 0.75	*α* = 1
2	2.504	0.304	0.076	0.060	0.068	0.092
5	10.863	0.207	0.066	0.092	0.136	0.217
10	37.549	0.129	0.100	0.191	0.409	9.329
20	136.364	0.157	0.194	0.891	28.354	33.200
50	809.024	0.377	0.724	186.600	268.848	321.844
*ɛ* = 10%						
N	*α* = 0	*α* = 0.1	*α* = 0.2	*α* = 0.5	*α* = 0.75	*α* = 1
2	8.910	2.493	0.285	0.066	0.073	0.096
5	39.015	2.203	0.089	0.098	0.142	0.240
10	133.655	0.404	0.107	0.207	0.474	9.746
20	493.386	0.182	0.203	1.249	28.467	33.925
50	2922.483	0.400	0.772	189.327	273.664	327.170
*ɛ* = 20%						
N	*α* = 0	*α* = 0.1	*α* = 0.2	*α* = 0.5	*α* = 0.75	*α* = 1
2	28.470	18.606	7.524	0.106	0.102	0.115
5	124.702	44.374	0.327	0.113	0.168	0.272
10	429.087	17.016	0.128	0.232	0.592	10.423
20	1576.600	0.335	0.229	3.460	29.091	34.815
50	9334.156	0.453	0.887	190.613	282.404	348.252

When there is no contamination, the MLE (*α* = 0) performs the best, whatever the dimension *N*. On the other hand, the estimations obtained with the minimum pseudodistance estimators in this case are very close to those provided by the MLE, when *α* is not far from zero (for example *α* = 0.1 and *α* = 0.2). In the presence of contamination, the minimum pseudodistance estimators give much better results than the MLE, in all considered cases. In most cases, the choices *α* = 0.1, *α* = 0.2 provide the best results in terms of robustness. In the meantime, these choices correspond to estimation procedures with high asymptotic relative efficiency, according to the results from Table 1. When the contamination is more pronounced, i.e. *ɛ* = 10% or *ɛ* = 20%, and the dimension *N* is low, i.e. *N* = 2, the choices *α* = 0.5 and *α* = 0.75 provide better robust estimates, but the asymptotic relative efficiencies of the corresponding estimation procedures are too low. Thus, values of *α* close to zero, such as *α* = 0.1, *α* = 0.2, represent choices that offer an equilibrium between robustness and efficiency. We notice that, even for highly multivariate data (*N* = 50 in our examples), when the number of observations is sufficiently high, the minimum pseudodistance estimators with *α* = 0.1, *α* = 0.2 give excellent results both in the case of clean data, as well as in case of contamination. The simulation results presented in Tables 2 and 3 show that increasing sample size leads to improved estimations.

Moreover, we present an example for highly correlated data. Here we considered the normal distribution $N_{N}(\mu ,\Sigma )$ with *N* = 5, *μ* = **0**, Σ the matrix with the diagonal terms 2 and the other entries 1. The contaminating distribution is $N_{N}(\mu_{c},\Sigma_{c})$, where *μ*_*c*_ = **4** and Σ_*c*_ = Σ, the contamination level being *ɛ* ∈ {0, 0.1, 0.2}. In this case, the mean square errors of the estimations are given in Table 4, the number of considered samples being again *n*_*s*_ = 1000. These results show that even in the case when the correlation level of the data is high and the data are contaminated, the minimum pseudodistance estimators give significantly better results than the MLE, the best results being obtained for *α* = 0.75 and *α* = 1.

**Table 4:** Simulation based estimates of the mean square error, for highly correlated data, *T* = 100 ⋅ *N*.

	*α* = 0	*α* = 0.1	*α* = 0.2	*α* = 0.5	*α* = 0.75	*α* = 1
*ɛ* = 0%	0.1999	0.2033	0.2281	0.3311	0.5232	0.8612
*ɛ* = 10%	32.3561	23.0288	13.9551	1.7271	0.9666	1.1272
*ɛ* = 20%	101.7404	97.5348	88.9003	34.6433	5.3518	2.0940

## Application for financial data

### In-sample analysis

**Fig 2:**
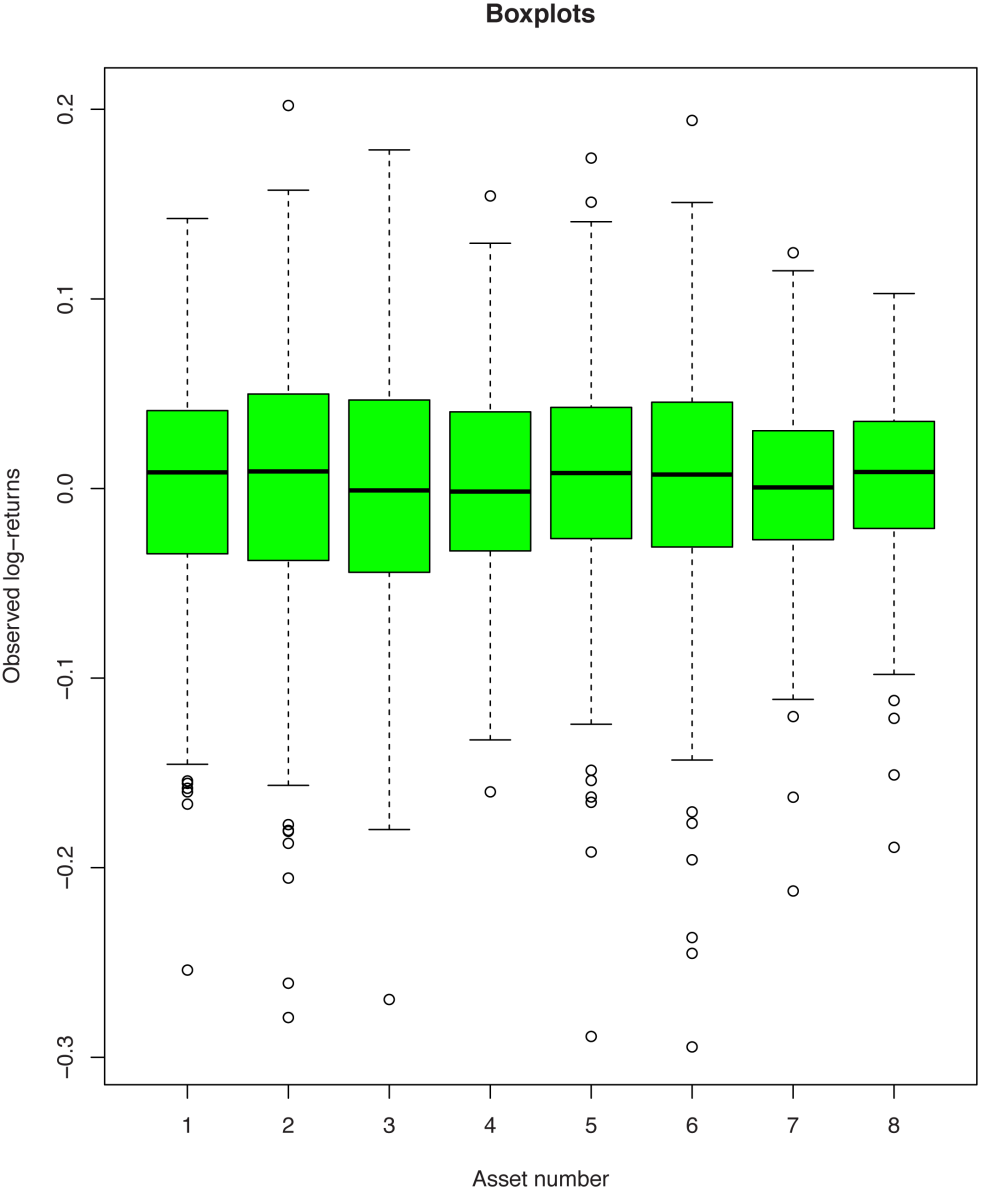
Boxplots for monthly log-returns corresponding to the MSCI Indexes. (1: France, 2: Germany, 3: Italy, 4: Japan, 5: Pacific ex JP, 6: Spain, 7: United Kingdom, 8: USA.)

We analyze 172 monthly log-returns of 8 MSCI Indexes (France, Germany, Italy, Japan, Pacific Ex JP, Spain, United Kingdom and USA) from January 1998 to April 2012 with the aim to construct robust and efficient portfolios. The data are provided by MSCI (see [31]). Boxplots for these data are presented in Fig 2.

**Fig 3:**
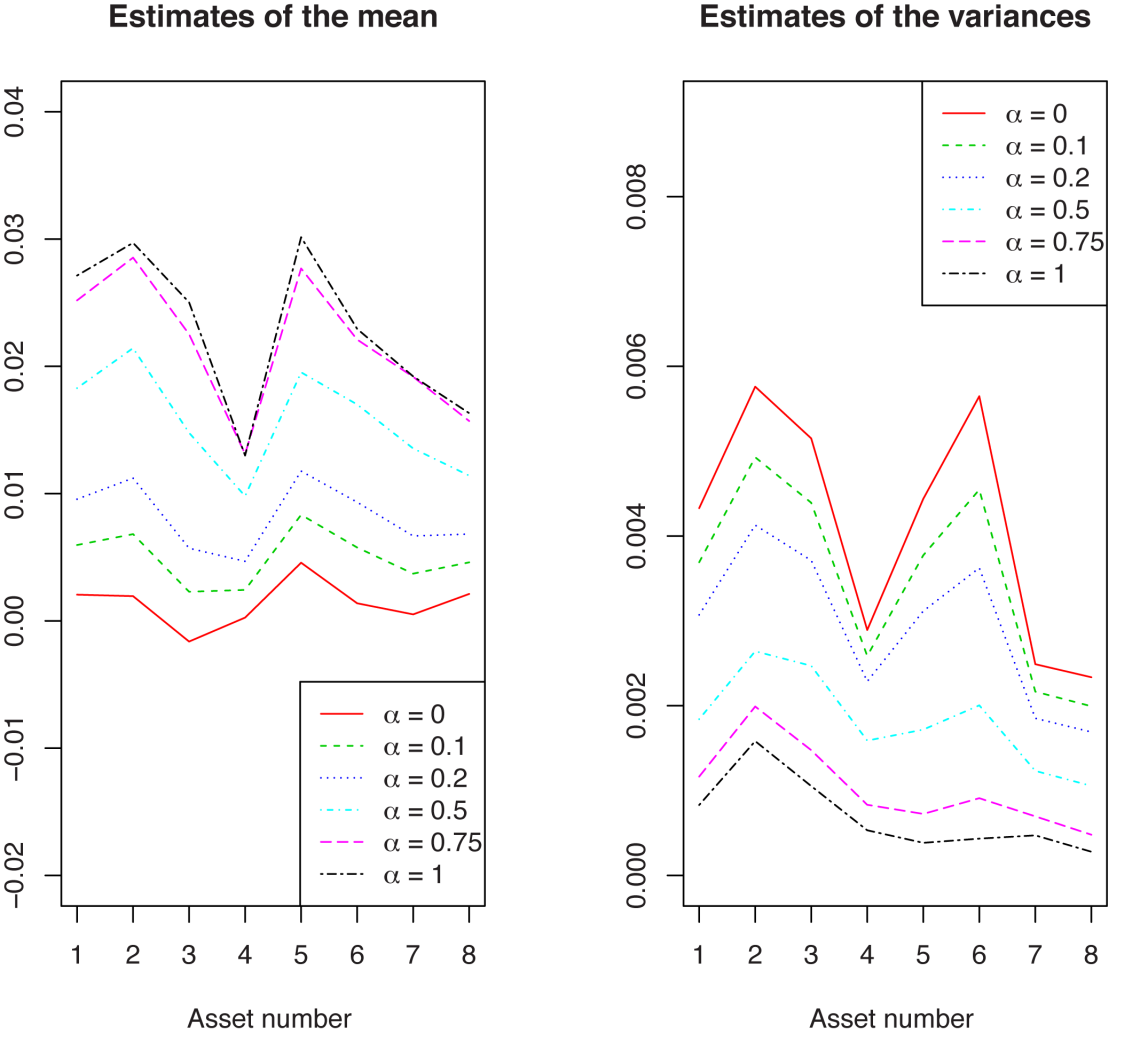
Expected returns estimates (left) and variance estimates (right) for the 8 MSCI Indexes. (1: France, 2: Germany, 3: Italy, 4: Japan, 5: Pacific ex JP, 6: Spain, 7: United Kingdom, 8: USA.)

For these indexes, estimates of the expected return and of the variance are represented in Fig 3. Note that the estimates of the expected returns obtained with the minimum pseudodistance estimators are larger than the maximum likelihood ones. In the meantime, the minimum pseudodistance estimates of the variances are smaller than those provided by the MLE.

**Fig 4:**
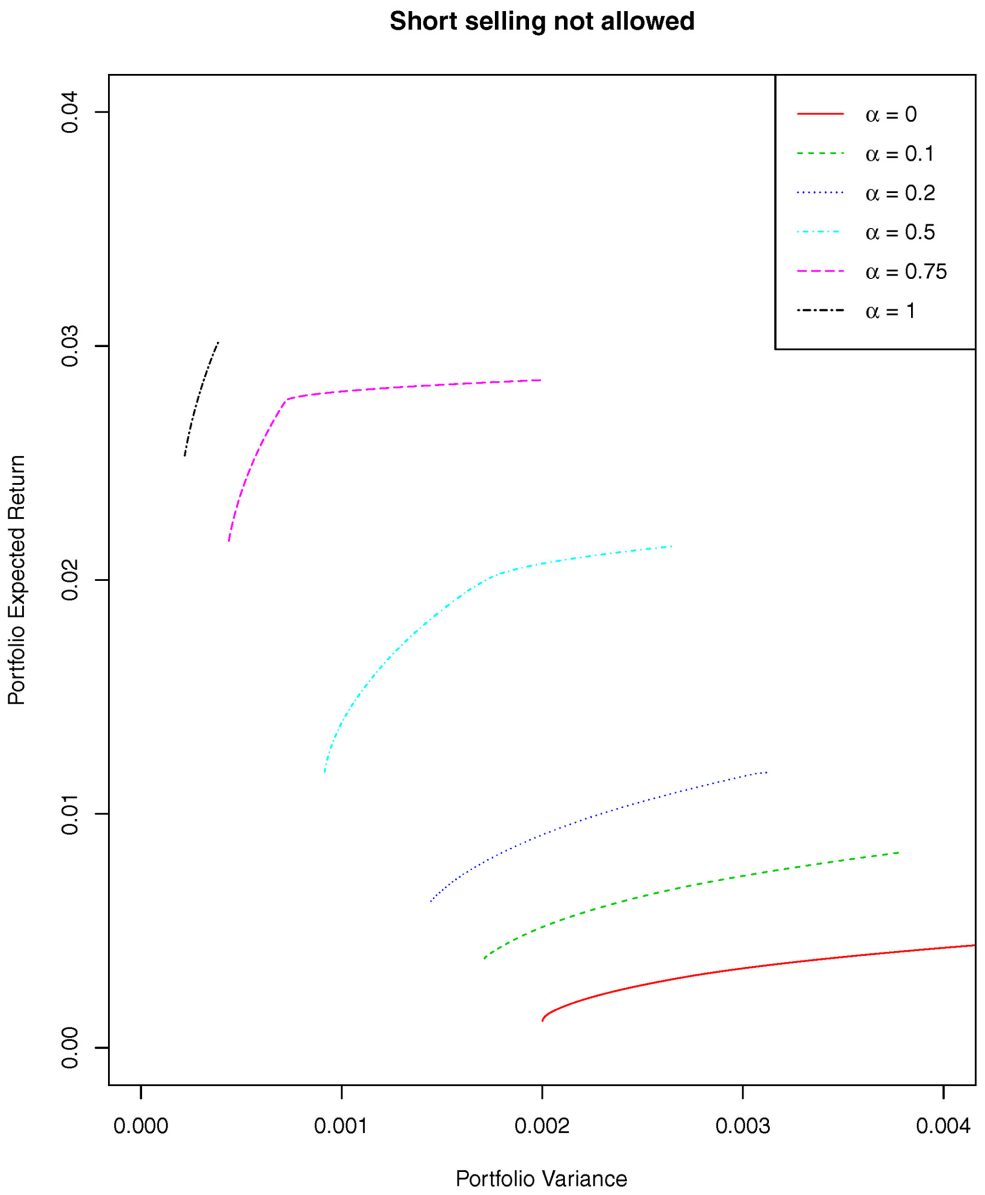
Mean-variance efficient frontiers.

Estimates of the mean vector and of the covariance matrix computed with different minimum pseudodistance estimators are used to determine efficient frontiers. In Fig 4 we plot efficient frontiers for the case “short selling not allowed”. Similar results can be obtained in the case “short selling allowed”. In both cases, the frontiers based on the minimum pseudodistance estimations dominate those based on the classical maximum likelihood estimations, yielding portfolios with larger expected returns and smaller risks. Thus, the robust estimates reduce the volatility effects which typically affects the results of the traditional approaches.

The next step in our analysis is to identify the influential observations which are responsible for the shift of the efficient frontier. We perform this study in the case “short selling”. In this sense, we use the data influence measure (DIM) as diagnostic tool (see [3]). This is defined as the Euclidian norm of the influence function of the estimator of weights based on maximum likelihood estimators of *μ* and Σ. More precisely,

$$\begin{array}{c} \text{DIM}\left(x,\hat{p^{*}}\right)={\left[\text{IF}{\left(x;p^{*},P_{\mu ,\Sigma }\right)}^{⊤}\text{IF}\left(x;p^{*},P_{\mu ,\Sigma }\right)\right]}^{1/2} \end{array}$$

where IF(*x*; p*, *P*_*μ*,Σ_) is given by Eq (27) with IF(*x*; *μ*, *P*_*μ*,Σ_) and IF(*x*; Σ, *P*_*μ*,Σ_) given by the formulas (18) and (19) in the case *α* = 0. In order to compute DIM, the true parameters values *μ*, Σ, p* have to be known. In practice, these parameters should be estimated in a robust way, such that DIM is not affected by the outlying observations it is supposed to detect.

**Fig 5:**
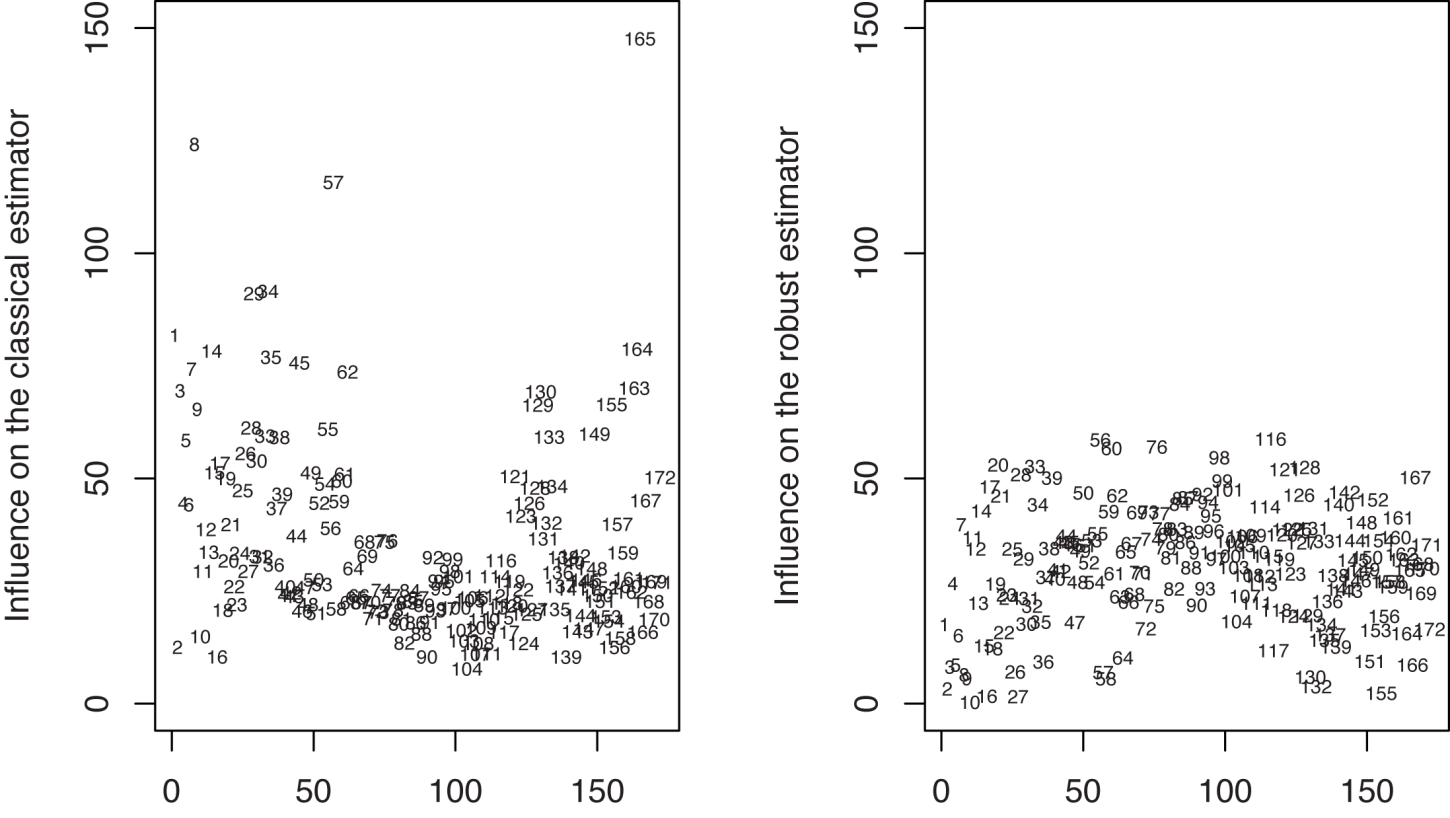
The influence of the observations on the classical/robust estimator of portfolio weights.

In Fig 5 (left hand side) we represent the influence of each of the 172 observations on the estimator of the optimal portfolio weights based on maximum likelihood estimators of *μ* and Σ. Since DIM is related to a specific portfolio on the efficient frontier, we made a choice, namely the level of the portfolio variance has been set to 0.005. This choice corresponds to *λ* = 3.85. The necessary robust estimates of *μ*, Σ, p* have been obtained with minimum pseudodistance estimators corresponding to *α* = 0.2. The most influential observations as detected by DIM correspond to negative economic events associated with known financial crisis periods: 1998 Russian financial crisis (August 1998), “dot-com crash” of 2000–2002 and 2007–2012 global financial crisis. On the other hand, the influence of these observations is substantially reduced when using robust procedures. This fact can be seen in the right hand side of Fig 5 where we represent the influence of each of the 172 observations on the robust estimator of the optimal portfolio weights based on the minimum pseudodistance estimators of *μ* and Σ corresponding to *α* = 0.2. Reducing the influence of outlying observations leads to optimal portfolios with higher returns and smaller variances.

### Out-of-sample evaluation

In this subsection, we illustrate the out-of-sample stability of the proposed portfolios. For this purpose we use the same empirical data set as in the preceding subsection. The evaluated portfolios are: optimal mean-variance portfolios using the classical MLE or minimum pseudodistance estimators (MPE) or S-estimators, minimum-variance portfolios using the classical MLE or minimum pseudodistance estimators or S-estimators, as well as the equally-weighted portfolio. The S-estimators are well known robust estimators of multivariate location and covariance, allowing the flexibility of choosing the breakdown point ɛ*, which is the amount of data deviating from the reference model that an estimator can accept while giving meaningful information (see for example [32]). We considered in our study S-estimators based on the Tukey’s biweight function with breakdown point ɛ* = 0.25 or ɛ* = 0.5. For the optimal mean-variance portfolios we used *λ* = 3.85, as in the preceding section, but similar results can be obtained for other finite values of *λ*, too. The minimum-variance portfolios correspond to *λ* = ∞, as it is known. We consider both cases, when short selling is allowed or not.

We compare the out-of-sample empirical performance of portfolios using three measures: turnover, the out-of-sample portfolio variance and the out-of-sample Sharpe ratio. For this we apply a “rolling-horizon” procedure as in [7]. First, we choose a window over which to perform the estimation. We denote the length of the estimation window by *τ* < *T*, where *T* is the size of the entire data set. For our examples, we use an estimation window of *τ* = 100 data points, *T* being 172. Then, using the data in the first estimation window, we compute the weights for the considered portfolios. We repeat this procedure for the next window, by including the data for the next month and dropping the data for the earliest month. We continue doing this until the end of the data set is reached. At the end of this process, we have generated *T* − *τ* portfolio weight vectors for each strategy, that is the vectors $p_{t}^{k}$ for *t* ∈ {*τ*, …, *T* − 1}, *k* denoting the strategy. For a strategy *k*, let $p_{j,t}^{k}$ denotes the portfolio weight in asset *j* chosen at time *t*, $p_{j,t+}^{k}$ the portfolio weight in asset *j* before rebalancing but at *t*+1 (considering the change in prices from *t* to *t*+1) and $p_{j,t+1}^{k}$ the portfolio weight in asset *j* at time *t*+1, after rebalancing. The portfolio turnover is defined as

$$\begin{array}{c} \text{Turnover}=\frac{1}{T-\tau -1}\sum_{t=\tau }^{T-1}\sum_{j=1}^{N}\left|p_{j,t+1}^{k}-p_{j,t+}^{k}\right|. \end{array}$$

The weights $p_{j,t+}^{k}$ are computed using the formula

$$\begin{array}{c} p_{j,t+}^{k}=\frac{1+X_{j}^{t+1}}{1+{\left(X^{t+1}\right)}^{⊤}p_{t}^{k}}\cdot p_{j,t}^{k} \end{array}$$

*X* ^*t*+1^ representing the data at the time *t* + 1. The out-of-sample return at time *t* + 1, corresponding to the strategy *k*, is defined as ${\left(p_{t}^{k}\right)}^{⊤}X^{t+1}$. For each strategy *k*, using these out-of-sample returns, the out-of-sample variance is defined by

$$\begin{array}{c} {\left({\hat{\sigma }}^{k}\right)}^{2}=\frac{1}{T-\tau -1}\sum_{t=\tau }^{T-1}{\left({\left(p_{t}^{k}\right)}^{⊤}X^{t+1}-{\hat{\mu }}^{k}\right)}^{2}\;\;\;\text{with}\;\;\;{\hat{\mu }}^{k}=\frac{1}{T-\tau }\sum_{t=\tau }^{T-1}{\left(p_{t}^{k}\right)}^{⊤}X^{t+1} \end{array}$$

and the Sharpe ratio is defined by ${\hat{SR}}^{k}=\frac{{\hat{\mu }}^{k}}{{\hat{\sigma }}^{k}}.$

In Tables 5 and 6 we report the turnover, the out-of-sample variance and the Sharpe ratio for optimal mean-variance portfolios (with *λ* = 3.85) and for minimum-variance portfolios in the case “short selling allowed”, respectively in the case “short selling not allowed”. For estimation of the mean and covariance we considered the classical MLE, minimum pseudodistance estimators for several values of *α*, as well as S-estimators. We note that the portfolios that minimize the variance have better turnover than the portfolios that optimize the trade-off between mean and variance, confirming the fact that the policies that ignore estimates of the expected returns lead to better stability results in the out-of-sample analysis. The robust portfolios using minimum pseudodistance estimators attain higher Sharpe ratios and lower turnover than the traditional portfolios using MLE, the influence of asset returns that deviate from normality being more reduced.

**Table 5:** Turnover, out-of-sample portfolio variance and Sharpe ratio for optimal mean-variance portfolios (Mean-var) and minimum-variance portfolios (Min-var), corresponding to different estimators of mean and covariance, in the case “short selling allowed”.

	Mean-var			Min-var		
Estimators	Turnover	${\left({\hat{\sigma }}^{k}\right)}^{2}$	${\hat{SR}}^{k}$	Turnover	${\left({\hat{\sigma }}^{k}\right)}^{2}$	${\hat{SR}}^{k}$
MLE	1.5665	0.0105	0.0368	0.2507	0.0021	-0.0378
MPE, *α* = 0.1	1.4717	0.0161	0.0727	0.2109	0.0020	-0.0282
MPE, *α* = 0.2	1.9083	0.0292	0.0880	0.1838	0.0019	-0.0322
MPE, *α* = 0.25	2.4381	0.0400	0.0911	0.1753	0.0019	-0.0367
SE, ɛ* = 0.25	1.4119	0.0126	0.0631	0.2283	0.0020	-0.0291
SE, ɛ* = 0.5	1.5506	0.0218	0.0878	0.1966	0.0019	-0.0302

**Table 6:** Turnover, out-of-sample portfolio variance and Sharpe ratio for optimal mean-variance portfolios (Mean-var) and minimum-variance portfolios (Min-var), corresponding to different estimators of mean and covariance, in the case “short selling not allowed”.

	Mean-var			Min-var		
Estimators	Turnover	${\left({\hat{\sigma }}^{k}\right)}^{2}$	${\hat{SR}}^{k}$	Turnover	${\left({\hat{\sigma }}^{k}\right)}^{2}$	${\hat{SR}}^{k}$
MLE	0.2068	0.0054	0.0032	0.0519	0.0025	-0.0304
MPE, *α* = 0.1	0.1510	0.0060	0.0186	0.0405	0.0025	-0.0264
MPE, *α* = 0.2	0.1475	0.0059	0.0170	0.0370	0.0025	-0.0220
MPE, *α* = 0.25	0.1562	0.0059	0.0138	0.0364	0.0026	-0.0199
MPE, *α* = 0.3	0.1594	0.0059	0.0129	0.0364	0.0026	-0.0184
MPE, *α* = 0.5	0.1084	0.0058	0.0254	0.0363	0.0026	-0.0148
MPE, *α* = 0.75	0.0235	0.0060	0.0319	0.0511	0.0028	-0.0050
MPE, *α* = 1	0.0143	0.0060	0.0345	0.1219	0.0028	-0.0259
SE, ɛ* = 0.25	0.1771	0.0058	0.0125	0.0435	0.0025	-0.0273
SE, ɛ* = 0.5	0.1320	0.0059	0.0173	0.0384	0.0026	-0.0224

When short selling is allowed, the mean-variance portfolios with the best turnover are those corresponding to the S-estimators with ɛ* = 0.25, respectively to the minimum pseudodistance estimators with *α* = 0.1, the Sharpe ratios of these portfolios being very close. When short selling is not allowed, the best mean-variance portfolios in terms of turnover and Sharpe ratio are those corresponding to the minimum pseudodistance estimators with *α* = 0.75 and *α* = 1. In the case of minimum-variance portfolios, the best turnover is obtained for minimum pseudodistance estimators (*α* = 0.2, 0.25, 0.3, 0.5) and the best Sharpe ratio is obtained for the minimim pseudodistance estimators with *α* = 0.75, both in the case “short selling not allowed”.

We also compare our results with those corresponding to the equally-weighted portfolio which is known to have a good out-of-sample behavior, even in the case of contaminated data. For our data set and this portfolio, we obtained: Turnover = 0.02234, ${\left({\hat{\sigma }}_{k}\right)}^{2}=0.0025$ and ${\hat{SR}}^{k}=-0.0272$. Note that, among all the considered portfolios, the equally-weighted portfolio has the smallest turnover. In the meantime, all the minimum-variance portfolios using minimum pseudodistance estimators, in the case “short selling not allowed”, have better Sharpe ratio than the equally-weighted portfolio. For example, the minimum-variance portfolio using minimum pseudodistance estimators with *α* = 0.5 represents a good choice in terms of turnover and Sharpe ratio.

**Fig 6:**
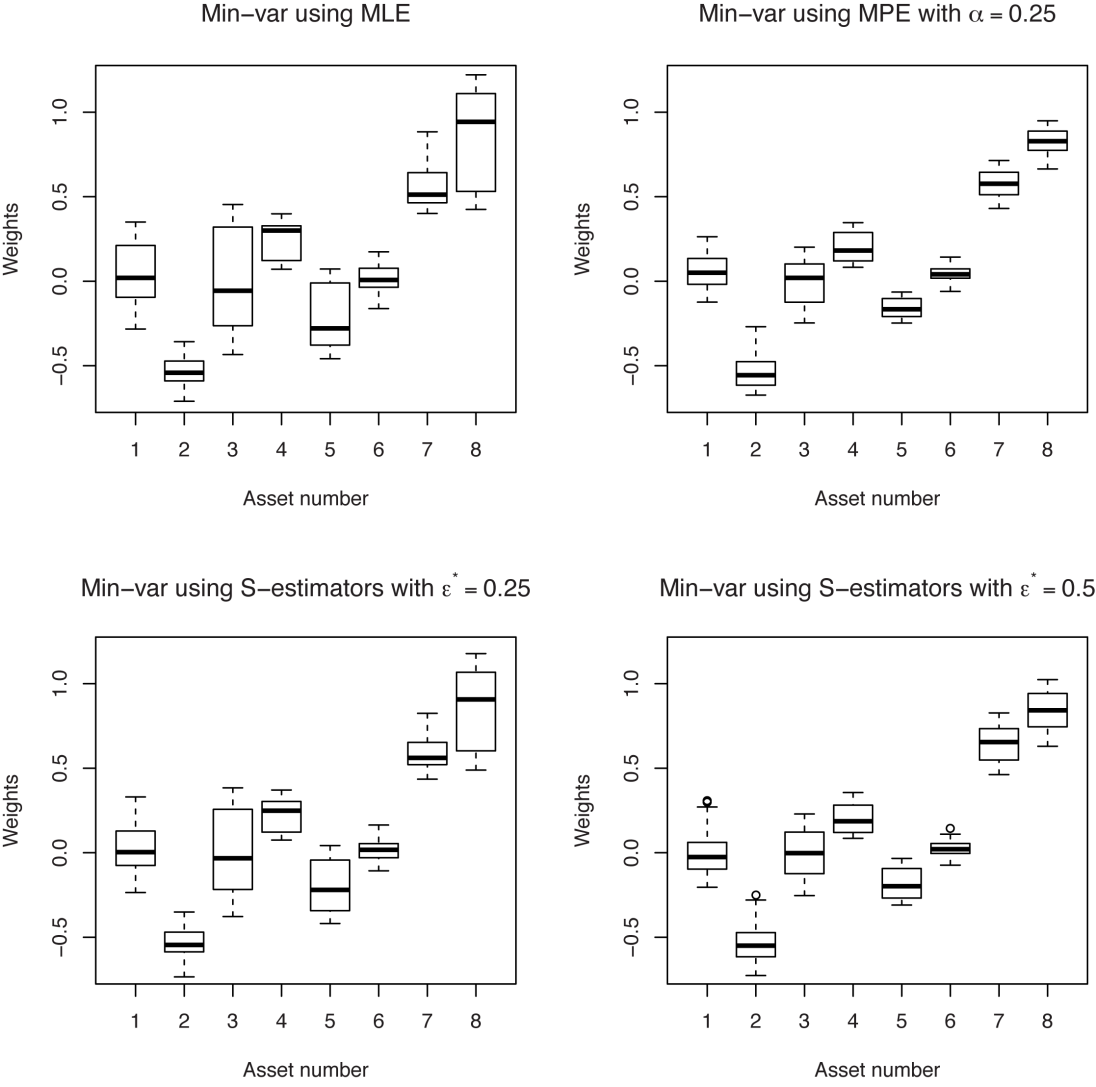
Boxplots of the weights corresponding to minimum-variance portfolios. Different estimators of the covariance matrix are used, in the case “short selling allowed”.

**Fig 7:**
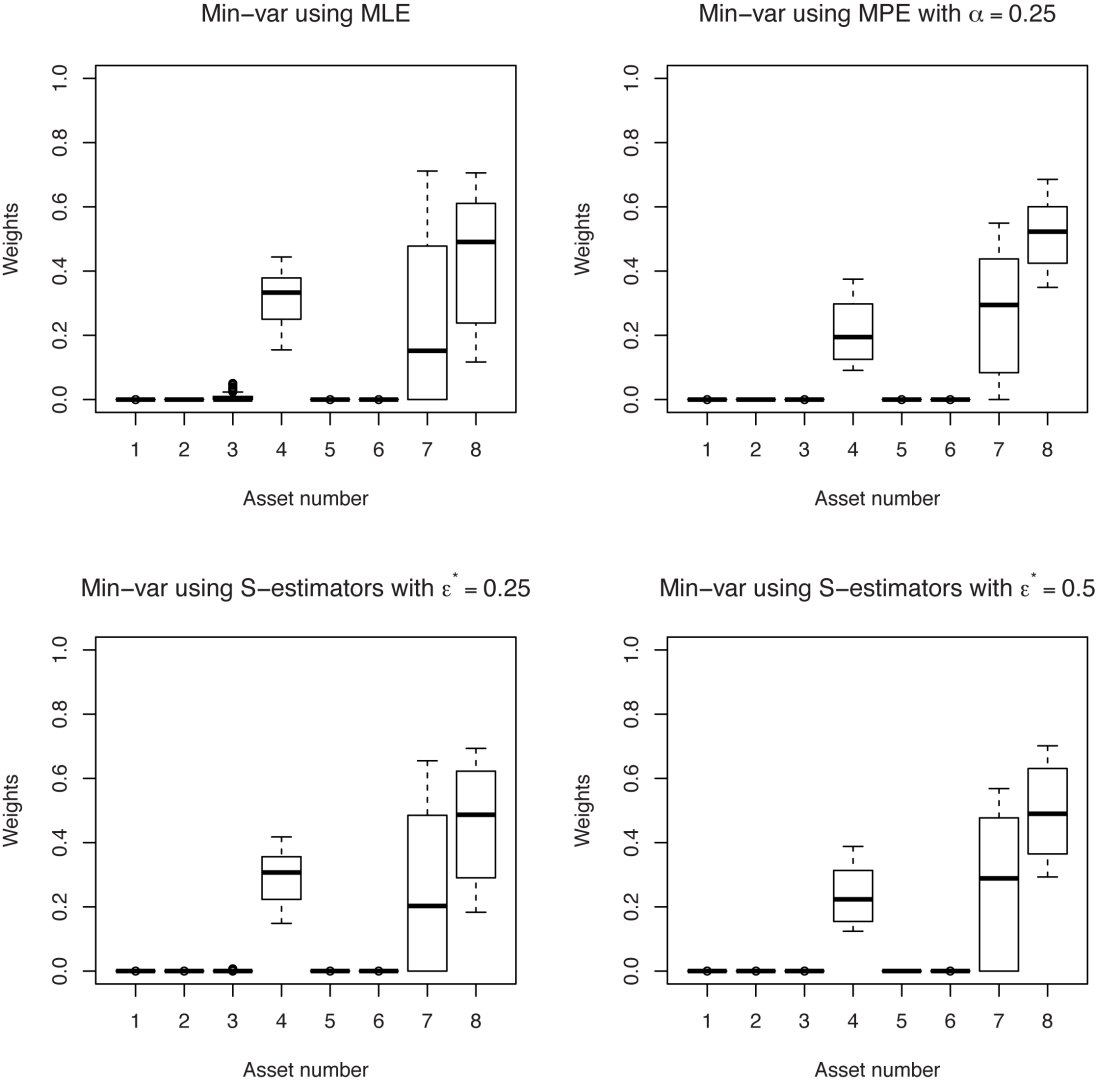
Boxplots of the weights corresponding to minimum-variance portfolios. Different estimators of the covariance matrix are used, in the case “short selling not allowed”.

The boxplots of portfolio weights give a graphical representation of the stability of the different portfolio policies. By applying the “rolling-horizon” procedure, we obtain *T* − *τ* portfolio weight vectors for each strategy. In Figs 6 and 7, each boxplot represent the variability of the weight assigned to a particular asset in a minimum-variance portfolio. As it can be seen, the robust minimum-variance portfolios based on minimum pseudodistance estimators are characterized by a better out-of-sample stability than the classical minimum-variance portfolios. We choose for these examples *α* = 0.25, but we obtained similar results for other values of *α*, too. Also, in this example, the weights of the minimum-variance portfolio based on minimum pseudodistance estimators are more stable than the weights of the minimum-variance portfolio based on S-estimators (although the out-of-sample behaviors of the portfolios based on these two type of estimators are very close).

Our theoretical and numerical results show that the optimal portfolios based on minimum pseudodistance estimators are much more stable to extreme events than those obtained by plugging-in the MLEs and compare well with other optimal robust portfolios. When *α* is not far from 0, the minimum pseudodistance estimators of *μ* and Σ combine robustness with high efficiency and these qualities are transferred to the portfolio weights estimator. The numerical results based on simulations or real data show that *α* = 0.2, 0.25 represent good choices in terms of robustness and efficiency, but also higher values of *α* lead to good results in some situations. All these aspects recommend the new procedure as a viable alternative to existing robust portfolio selection methods.

## Supporting Information

S1 Appendix Appendix. Codes of the Figures.
(DOC)
