# Supplementary material for: Robust Portfolio Optimization Using Pseudodistances
Source: PLoS One. 2015 Oct 15;10(10):e0140546. doi: 10.1371/journal.pone.0140546 (PMC4607458; doi:10.1371/journal.pone.0140546)
Supplement: S1 Appendix — Codes of the Figures. (DOC) [file pone.0140546.s001.doc]

**Code for Figure 1**

alpha <- 0.5

x<-seq(-6,6,length=50)

y<-seq(-6,6,length=50)

IF<-matrix(0,50,50)

for(i in 1:50)

{

for(j in 1:50)

{

IF[i,j] <- ((alpha+1)^3)*(x[i]*y[j])*(exp(-(alpha/2)*(x[i]^2+y[j]^2)))

}}

**Codes for Figures 2 and 5**

dport <- read.csv(file="C:/sleoni/Aida2011/Real Data/DatiInsieme.csv", sep=";",dec=",", header=TRUE)

library(MASS)

nsam <- 172

dN <- 8

matrd<-array(0, c(nsam,dN))

matrd[,1]<-dport$FRANCE

matrd[,2]<-dport$GERMANY

matrd[,3]<-dport$ITALY

matrd[,4]<-dport$JAPAN

matrd[,5]<-dport$PACIFIC.EX.JAPAN

matrd[,6]<-dport$SPAIN

matrd[,7]<-dport$UNITED.KINGDOM

matrd[,8]<-dport$USA

# Fig. 2

boxplot(matrd, ylab="Observed log-returns", main="Boxplots", xlab = "Asset number",col="green")

alpha <- 0.2

muhat <- array(0,c(dN))

sigmahat <- array(0,c(dN, dN))

x <- array(0, c(nsam,dN))

x <- matrd

# initial values for mu and sigma

muinit <- apply(x,2,mean)

sigmainit <- cov(x)

stepmax <- 800

estmu <- array(0, c( dN, stepmax+1 ))

estsig <- array(0, c (dN, dN, stepmax+1 ))

investsig <- array(0, c (dN, dN, stepmax+1 ))

weiwj <- array(0, c(nsam, stepmax+1))

numweiwj <- array(0, c(nsam, stepmax+1))

denweiwj <- array(0, c( stepmax+1))

weiwjxj1 <- array(0, c( nsam, dN, stepmax+1))

sigsumstep2 <- array(0, c( nsam, dN, dN, stepmax+1))

ste <- 1

estmu[,1] <- muinit

estsig[,,1] <- sigmainit

ste <- 2

investsig[,,1] <- solve(estsig[,,1])

# computation wj

xjdifmu <- array(0, c(nsam, dN))

for(j in 1: nsam)

{

xjdifmu[j, ] <- x[j,]-estmu[,1]

numweiwj[j,1] <- exp(- alpha * t(xjdifmu[j,]) %*% (investsig[,,1]) %*% xjdifmu[j, ] /2)

}

denweiwj[1] <- sum(numweiwj[,1])

for(j in 1: nsam)

{

weiwj[j,1] <- numweiwj[j,1]/denweiwj[1]

for(dd in 1:dN){

weiwjxj1[j,dd,1] <- weiwj[j,1]*x[j,dd]

}

}

for(dd in 1:dN) {

estmu[dd,2] <- sum(weiwjxj1[,dd,1])

}

for(j in 1: nsam)

{

sigsumstep2[j,,,2] <- (alpha +1)* weiwj[j,1] * (x[j,]-estmu[,2]) %*% t(x[j,]-estmu[,2])

}

for(dd1 in 1:dN) {

for(dd2 in 1:dN) {

estsig[dd1, dd2, 2] <- sum(sigsumstep2[,dd1,dd2,2])

}

}

diffmu <- array(1, c(dN,stepmax+1))

for(dd in 1:dN)

{

diffmu[dd,ste]<-abs(estmu[dd,ste]-estmu[dd, ste-1])

}

diffsigma <- array(1, c(dN,dN,stepmax+1))

for(dd1 in 1:dN) {

for(dd2 in 1:dN) {

diffsigma[dd1, dd2, ste] <- abs(estsig[dd1,dd2,ste]-estsig[dd1,dd2, ste-1])

}

}

while( ( (max(diffmu[,ste])>0.00000000001) || ( max( diffsigma[,,ste] ) >0.00000000001 ) ) && (ste <= stepmax) )

{

determestsig <- det(estsig[,,ste])

if (determestsig <= 0.00000000001) {

for(dd in 1:dN){

estsig[dd,dd,ste]<- estsig[dd,dd,ste]+ 0.0001

}

}

determestsig <- det(estsig[,,ste])

investsig[,,ste] <- solve(estsig[,,ste])

xjdifmu <- array(0, c(nsam, dN))

for(j in 1: nsam)

{

xjdifmu[j, ] <- x[j,]-estmu[,ste]

numweiwj[j,ste] <- exp(- alpha * t(xjdifmu[j,]) %*% (investsig[,,ste]) %*% xjdifmu[j, ] /2)

}

denweiwj[ste] <- sum(numweiwj[,ste])

for(j in 1: nsam)

{

weiwj[j,ste] <- numweiwj[j,ste]/denweiwj[ste]

for(dd in 1:dN){

weiwjxj1[j,dd,ste] <- weiwj[j,ste]*x[j,dd]

}

}

ste <- ste+1

for(dd in 1:dN) {

estmu[dd,ste] <- sum(weiwjxj1[,dd,ste-1])

}

for(j in 1: nsam)

{

sigsumstep2[j,,,ste] <- (alpha +1)* weiwj[j,ste-1] * (x[j,]-estmu[,ste]) %*% t(x[j,]-estmu[,ste])

}

for(dd1 in 1:dN) {

for(dd2 in 1:dN) {

estsig[dd1, dd2, ste] <- sum(sigsumstep2[,dd1,dd2,ste])

}

}

for(dd in 1:dN)

{

diffmu[dd,ste]<-abs(estmu[dd,ste]-estmu[dd, ste-1])

}

for(dd1 in 1:dN) {

for(dd2 in 1:dN) {

diffsigma[dd1, dd2, ste] <- abs(estsig[dd1,dd2,ste]-estsig[dd1,dd2, ste-1])

}

}

}

muhat <- estmu[, ste]

sigmahat <-estsig[ , , ste]

#muhata02 <- array(0, c(8))

#sigmahata02 <- array(0, c(8,8))

muhata02 <- muhat

sigmahata02 <- sigmahat[,]

# Influence on the robust estimator

lamb <- 3.855

invsiga02 <- solve(sigmahata02)

vet1 <- array(1,dim=c(8,1))

denoma02 <- t(vet1) %*% invsiga02 %*% vet1

numa02x <- array(0, c(1))

eta02x <- array(0, c(1))

pal02x <- array(0, c(8,1))

for(i in 1:1)

{

numa02x[i] <- (t(vet1) %*% invsiga02 %*% muhata02 ) - lamb

eta02x[i] <- numa02x[i]/denoma02

pal02x[,i]<- (1/lamb)* invsiga02 %*% (muhata02 - vet1 %*% eta02x)

}

ifxmu <- function(x){

(sqrt(0.2+1))^(8+2)*(x-muhata02)%*% exp(-(0.2/2)*t(x-muhata02)%*% invsiga02 %*%(x-muhata02))

}

eifsig <- function(y){

exp(-(0.2/2)*t(y-muhata02)%*% invsiga02 %*%(y-muhata02))

}

ifxsig <- function(z){

(sqrt(0.2+1))^(8+4)*( ( (z-muhata02)%*% t(z-muhata02) ) - (1/(0.2+1))* sigmahata02 ) *drop(eifsig(y=z))

}

ifA <- function(yy){

-invsiga02 %*%ifxsig(z=yy)%*%pal02x

}

ifb <- function(yyy){

(ifxsig(z=yyy) %*% invsiga02 %*% muhata02 ) - ifxmu(x=yyy)

}

ifbb<-function(yyyy){

(drop(t(vet1) %*% invsiga02%*%ifb(yyy=yyyy))* vet1 )/drop(t(vet1)%*%invsiga02 %*% vet1)

}

ifc <- function(zz){

vet1*(drop(t(vet1)%*% invsiga02 %*% muhata02) -

lamb) * drop(t(vet1)%*% invsiga02 %*% ifxsig(z=zz) %*% invsiga02 %*% vet1 ) /(drop(t(vet1)%*%invsiga02 %*% vet1))^2

}

ifglob <- function(ee){

ifA(yy=ee)+(1/lamb)* invsiga02 %*%(ifxmu(x=ee)+ifbb(yyyy=ee)+ifc(zz=ee) )

}

DIM <- function(valore){

sqrt(drop(t(ifglob(ee=valore))%*%ifglob(ee=valore) ))

}

dataim<- array(0, c(172))

for(i in 1: 172)

{

dataim[i]<- DIM(matrd[i,])

}

# Influence on the classical estimator

lamb0 <- 3.855

ifxmu0 <- function(x){

(sqrt(0+1))^(8+2)*(x-muhata02)%*% exp(-(0/2)*t(x-muhata02)%*% invsiga02 %*%(x-muhata02))

}

eifsig0 <- function(y){

exp(-(0/2)*t(y-muhata02)%*% invsiga02 %*%(y-muhata02))

}

ifxsig0 <- function(z){

(sqrt(0+1))^(8+4)*( ( (z-muhata02)%*% t(z-muhata02) ) - (1/(0+1))* sigmahata02 ) *drop(eifsig0(y=z))

}

ifA0 <- function(yy){

-invsiga02 %*%ifxsig0(z=yy)%*%pal02x

}

ifb0 <- function(yyy){

(ifxsig0(z=yyy) %*% invsiga02 %*% muhata02 ) - ifxmu0(x=yyy)

}

ifbb0<-function(yyyy){

(drop(t(vet1) %*% invsiga02%*%ifb0(yyy=yyyy))* vet1 )/drop(t(vet1)%*%invsiga02 %*% vet1)

}

ifc0 <- function(zz){

vet1*(drop(t(vet1)%*% invsiga02 %*% muhata02) -

lamb0) * drop(t(vet1)%*% invsiga02 %*% ifxsig0(z=zz) %*% invsiga02 %*% vet1 ) /(drop(t(vet1)%*%invsiga02 %*% vet1))^2

}

ifglob0 <- function(ee){

ifA0(yy=ee)+(1/lamb0)* invsiga02 %*%(ifxmu0(x=ee)+ifbb0(yyyy=ee)+ifc0(zz=ee) )

}

DIM0 <- function(valore){

sqrt(drop(t(ifglob0(ee=valore))%*%ifglob0(ee=valore) ))

}

dataim0<- array(0, c(172))

for(i in 1: 172)

{

dataim0[i]<- DIM0(matrd[i,])

}

# Fig. 5

par(mfrow=c(1,2))

plot(dataim0, type="n", xlab=" " , ylab= "Influence on the classical estimator ", ylim=c(0,150))

text(dataim0, cex=.6)

plot(dataim, type="n", xlab=" ", ylab= "Influence on the robust estimator ", ylim=c(0,150))

text(dataim, cex=.6)

**Code for Figure 3**

dport <- read.csv(file="C:/sleoni/Aida2011/Real Data/DatiInsieme.csv", sep=";",dec=",", header=TRUE)

library(MASS)

nsam <- 172

dN <- 8

matrd<-array(0, c(nsam,dN))

matrd[,1]<-dport$FRANCE

matrd[,2]<-dport$GERMANY

matrd[,3]<-dport$ITALY

matrd[,4]<-dport$JAPAN

matrd[,5]<-dport$PACIFIC.EX.JAPAN

matrd[,6]<-dport$SPAIN

matrd[,7]<-dport$UNITED.KINGDOM

matrd[,8]<-dport$USA

alpha<- c(0,0.1,0.2,0.5,0.75,1)

x <- array(0, c(nsam,dN))

x <- matrd

# initial values for mu and sigma

muinit <- apply(x,2,mean)

sigmainit <- cov(x)

stepmax <- 800

muhatalpha <- array(0, c(dN,6))

sigmahatalpha <- array(0, c(dN,dN,6))

for(al in 1:6)

{

muhat <- array(0,c(dN))

sigmahat <- array(0,c(dN, dN))

estmu <- array(0, c( dN, stepmax+1 ))

estsig <- array(0, c (dN, dN, stepmax+1 ))

investsig <- array(0, c (dN, dN, stepmax+1 ))

weiwj <- array(0, c(nsam, stepmax+1))

numweiwj <- array(0, c(nsam, stepmax+1))

denweiwj <- array(0, c( stepmax+1))

weiwjxj1 <- array(0, c( nsam, dN, stepmax+1))

sigsumstep2 <- array(0, c( nsam, dN, dN, stepmax+1))

ste <- 1

estmu[,1] <- muinit

estsig[,,1] <- sigmainit

ste <- 2

investsig[,,1] <- solve(estsig[,,1])

# computation wj

xjdifmu <- array(0, c(nsam, dN))

for(j in 1: nsam)

{

xjdifmu[j, ] <- x[j,]-estmu[,1]

numweiwj[j,1] <- exp(- alpha[al] * t(xjdifmu[j,]) %*% (investsig[,,1]) %*% xjdifmu[j, ] /2)

}

denweiwj[1] <- sum(numweiwj[,1])

for(j in 1: nsam)

{

weiwj[j,1] <- numweiwj[j,1]/denweiwj[1]

for(dd in 1:dN){

weiwjxj1[j,dd,1] <- weiwj[j,1]*x[j,dd]

}

}

for(dd in 1:dN) {

estmu[dd,2] <- sum(weiwjxj1[,dd,1])

}

for(j in 1: nsam)

{

sigsumstep2[j,,,2] <- (alpha[al] +1)* weiwj[j,1] * (x[j,]-estmu[,2]) %*% t(x[j,]-estmu[,2])

}

for(dd1 in 1:dN) {

for(dd2 in 1:dN) {

estsig[dd1, dd2, 2] <- sum(sigsumstep2[,dd1,dd2,2])

}

}

diffmu <- array(1, c(dN,stepmax+1))

for(dd in 1:dN)

{

diffmu[dd,ste]<-abs(estmu[dd,ste]-estmu[dd, ste-1])

}

diffsigma <- array(1, c(dN,dN,stepmax+1))

for(dd1 in 1:dN) {

for(dd2 in 1:dN) {

diffsigma[dd1, dd2, ste] <- abs(estsig[dd1,dd2,ste]-estsig[dd1,dd2, ste-1])

}

}

while( ( (max(diffmu[,ste])>0.00000000001) || ( max( diffsigma[,,ste] ) >0.00000000001 ) ) && (ste <= stepmax) )

{

determestsig <- det(estsig[,,ste])

if (determestsig <= 0.00000000001) {

for(dd in 1:dN){

estsig[dd,dd,ste]<- estsig[dd,dd,ste]+ 0.0001

}

}

determestsig <- det(estsig[,,ste])

investsig[,,ste] <- solve(estsig[,,ste])

xjdifmu <- array(0, c(nsam, dN))

for(j in 1: nsam)

{

xjdifmu[j, ] <- x[j,]-estmu[,ste]

numweiwj[j,ste] <- exp(- alpha[al] * t(xjdifmu[j,]) %*% (investsig[,,ste]) %*% xjdifmu[j, ] /2)

}

denweiwj[ste] <- sum(numweiwj[,ste])

for(j in 1: nsam)

{

weiwj[j,ste] <- numweiwj[j,ste]/denweiwj[ste]

for(dd in 1:dN){

weiwjxj1[j,dd,ste] <- weiwj[j,ste]*x[j,dd]

}

}

ste <- ste+1

for(dd in 1:dN) {

estmu[dd,ste] <- sum(weiwjxj1[,dd,ste-1])

}

for(j in 1: nsam)

{

sigsumstep2[j,,,ste] <- (alpha[al] +1)* weiwj[j,ste-1] * (x[j,]-estmu[,ste]) %*% t(x[j,]-estmu[,ste])

}

for(dd1 in 1:dN) {

for(dd2 in 1:dN) {

estsig[dd1, dd2, ste] <- sum(sigsumstep2[,dd1,dd2,ste])

}

}

for(dd in 1:dN)

{

diffmu[dd,ste]<-abs(estmu[dd,ste]-estmu[dd, ste-1])

}

for(dd1 in 1:dN) {

for(dd2 in 1:dN) {

diffsigma[dd1, dd2, ste] <- abs(estsig[dd1,dd2,ste]-estsig[dd1,dd2, ste-1])

}

}

}

muhat <- estmu[, ste]

sigmahat <-estsig[ , , ste]

muhatalpha[,al]<- muhat

sigmahatalpha[,,al] <- sigmahat[,]

}

# Fig. 3 (left)

plot(muhatalpha[,1], type="l", lty = 1, ylim=c(-0.02, 0.04),col=2, ylab= " ", xlab= "Asset number ",main= "Estimates of the mean")

par(new=T)

plot(muhatalpha[,2], type="l", lty=2, ylim=c(-0.02, 0.04), col=3, ylab= " ", xlab= " ")

par(new=T)

plot(muhatalpha[,3], type="l", lty = 3, ylim=c(-0.02, 0.04), col=4, ylab= " ", xlab= " ")

par(new=T)

plot(muhatalpha[,4], type="l", lty=4, ylim=c(-0.02, 0.04), col=5, ylab= " ", xlab= " ")

par(new=T)

plot(muhatalpha[,5], type="l", lty=5, ylim=c(-0.02, 0.04), col=6, ylab= " ", xlab= " ")

par(new=T)

plot(muhatalpha[,6], type="l", lty=6, ylim=c(-0.02, 0.04), col=1, ylab= " ", xlab= " ")

legend("bottomright", c(expression(paste(alpha, " = 0 ")),expression(paste(alpha, " = 0.1 ")),expression(paste(alpha, " = 0.2 ")), expression(paste(alpha, " = 0.5 ")), expression(paste(alpha, " = 0.75 ")), expression(paste(alpha, " = 1 "))), lty=c(1:6), col=c(2,3,4,5,6,1) )

vara0 <- array(0, c(8))

for(i in 1:8){

vara0[i]<- sigmahatalpha[i,i,1]

}

vara01 <- array(0, c(8))

for(i in 1:8){

vara01[i]<- sigmahatalpha[i,i,2]

}

vara02 <- array(0, c(8))

for(i in 1:8){

vara02[i]<- sigmahatalpha[i,i,3]

}

vara05 <- array(0, c(8))

for(i in 1:8){

vara05[i]<- sigmahatalpha[i,i,4]

}

vara075 <- array(0, c(8))

for(i in 1:8){

vara075[i]<- sigmahatalpha[i,i,5]

}

vara1 <- array(0, c(8))

for(i in 1:8){

vara1[i]<- sigmahatalpha[i,i,6]

}

# Fig. 3 (right)

plot(vara0, type="l", lty = 1, ylim=c(0, 0.009), col=2, ylab= " ", xlab= "Asset number ",main= "Estimates of the variances")

par(new=T)

plot(vara01, type="l", lty = 2, ylim=c(0, 0.009), col=3, ylab= " ", xlab= " ")

par(new=T)

plot(vara02, type="l", lty = 3, ylim=c(0, 0.009), col=4, ylab= " ", xlab= " ")

par(new=T)

plot(vara05, type="l", lty = 4, ylim=c(0, 0.009), col=5, ylab= " ", xlab= " ")

par(new=T)

plot(vara075, type="l", lty = 5, ylim=c(0, 0.009), col=6, ylab= " ", xlab= " ")

par(new=T)

plot(vara1, type="l", lty = 6, ylim=c(0, 0.009), col=1, ylab= " ", xlab= " ")

legend("topright", c(expression(paste(alpha, " = 0 ")),expression(paste(alpha, " = 0.1 ")),expression(paste(alpha, " = 0.2 ")), expression(paste(alpha, " = 0.5 ")), expression(paste(alpha, " = 0.75 ")), expression(paste(alpha, " = 1 "))),lty = c(1:6), col=c(2,3,4,5,6,1))

**Code for Figure 4**

# to run after Fig.3

library(quadprog)

lam <- seq(0.000000001, 100, by=0.001)

vet1 <- array(1,dim=c(8,1))

# alpha = 0

invsiga0 <- solve(sigmahatalpha[,,1])

denoma0 <- t(vet1) %*% invsiga0 %*% vet1

numa0 <- array(0, c(100000))

eta0 <- array(0, c(100000))

pal0n <- array(0, c(8,100000))

Dmatrice <-array(0, c(8,8,100000))

devecmu0 <- muhatalpha[,1]

Amatm <- cbind(t(array(1, dim = c(1,8))), diag(8))

bvec0 <- as.matrix(c(1, rep.int(0,8)))

for(i in 1:100000)

{

Dmatrice[,,i] <- lam[i] * sigmahatalpha[,,1]

pal0n[,i] <- solve.QP(Dmat= Dmatrice[,,i], dvec = devecmu0, Amat = Amatm , bvec = bvec0 , meq=1)$solution

numa0[i] <- (t(vet1) %*% invsiga0 %*% muhatalpha[,1] ) - lam[i]

eta0[i] <- numa0[i]/denoma0

}

reta0n <- array(0, c(100000))

sa0n <- array(0, c(100000))

for(i in 1:100000)

{

reta0n[i] <- t(pal0n[,i])%*%muhatalpha[,1]

sa0n[i] <- t(pal0n[,i]) %*% sigmahatalpha[,,1] %*% pal0n[,i]

}

plot(sa0n, reta0n, type="l", main= expression(paste(alpha, " = 0 ")), xlab ="Portfolio Variance", ylab="Portfolio Expected Return", xlim=c(0,0.004), ylim=c(0,0.02), col=3)

# alpha = 0.1

invsiga01 <- solve(sigmahatalpha[,,2] )

denoma01 <- t(vet1) %*% invsiga01 %*% vet1

numa01 <- array(0, c(100000))

eta01 <- array(0, c(100000))

pal01n <- array(0, c(8,100000))

Dmatrice <-array(0, c(8,8,100000))

devecmu01 <- muhatalpha[,2]

for(i in 1:100000)

{

Dmatrice[,,i] <- lam[i] * sigmahatalpha[,,2]

pal01n[,i] <- solve.QP(Dmat= Dmatrice[,,i], dvec = devecmu01, Amat = Amatm , bvec = bvec0 , meq=1)$solution

numa01[i] <- (t(vet1) %*% invsiga01 %*% muhatalpha[,2] ) - lam[i]

eta01[i] <- numa01[i]/denoma01

}

reta01n <- array(0, c(100000))

sa01n <- array(0, c(100000))

for(i in 1:100000)

{

reta01n[i] <- t(pal01n[,i])%*%muhatalpha[,2]

sa01n[i] <- t(pal01n[,i]) %*% sigmahatalpha[,,2] %*% pal01n[,i]

}

plot(sa01n, reta01n, type="l", main= expression(paste(alpha, " = 0.1 ")), xlab ="Portfolio Variance", ylab="Portfolio Expected Return", xlim=c(0,0.004), ylim=c(0,0.02), col=3)

# alpha = 0.2

invsiga02 <- solve(sigmahatalpha[,,3])

denoma02 <- t(vet1) %*% invsiga02 %*% vet1

numa02 <- array(0, c(100000))

eta02 <- array(0, c(100000))

pal02n <- array(0, c(8,100000))

Dmatrice <-array(0, c(8,8,100000))

devecmu <- muhatalpha[,3]

for(i in 1:100000)

{

Dmatrice[,,i] <- lam[i] * sigmahatalpha[,,3]

pal02n[,i] <- solve.QP(Dmat= Dmatrice[,,i], dvec = devecmu, Amat = Amatm , bvec = bvec0 , meq=1)$solution

numa02[i] <- (t(vet1) %*% invsiga02 %*% muhatalpha[,3] ) - lam[i]

eta02[i] <- numa02[i]/denoma02

}

reta02n <- array(0, c(100000))

sa02n <- array(0, c(100000))

for(i in 1:100000)

{

reta02n[i] <- t(pal02n[,i])%*%muhatalpha[,3]

sa02n[i] <- t(pal02n[,i]) %*% sigmahatalpha[,,3] %*% pal02n[,i]

}

plot(sa02n, reta02n, type="l", main= expression(paste(alpha, " = 0.2 ")), xlab ="Portfolio Variance", ylab="Portfolio Expected Return", xlim=c(0,0.004), ylim=c(0,0.02), col=3)

# alpha = 0.5

invsiga05 <- solve(sigmahatalpha[,,4] )

denoma05 <- t(vet1) %*% invsiga05 %*% vet1

numa05 <- array(0, c(100000))

eta05 <- array(0, c(100000))

pal05n <- array(0, c(8,100000))

Dmatrice05 <-array(0, c(8,8,100000))

devecmu05 <- muhatalpha[,4]

for(i in 1:100000)

{

Dmatrice05[,,i] <- lam[i] * sigmahatalpha[,,4]

pal05n[,i] <- solve.QP(Dmat= Dmatrice05[,,i], dvec = devecmu05, Amat = Amatm , bvec = bvec0 , meq=1)$solution

numa05[i] <- (t(vet1) %*% invsiga05 %*% muhatalpha[,4] ) - lam[i]

eta05[i] <- numa05[i]/denoma05

}

reta05n <- array(0, c(100000))

sa05n <- array(0, c(100000))

for(i in 1:100000)

{

reta05n[i] <- t(pal05n[,i])%*%muhatalpha[,4]

sa05n[i] <- t(pal05n[,i]) %*% sigmahatalpha[,,4] %*% pal05n[,i]

}

plot(sa05n, reta05n, type="l", main= expression(paste(alpha, " = 0.5 ")), xlab ="Portfolio Variance", ylab="Portfolio Expected Return", xlim=c(0,0.004), ylim=c(0,0.04), col=3)

# alpha = 0.75

invsiga075 <- solve(sigmahatalpha[,,5] )

denoma075 <- t(vet1) %*% invsiga075 %*% vet1

numa075 <- array(0, c(100000))

eta075 <- array(0, c(100000))

pal075n <- array(0, c(8,100000))

Dmatrice075 <-array(0, c(8,8,100000))

devecmu075 <- muhatalpha[,5]

for(i in 1:100000)

{

Dmatrice075[,,i] <- lam[i] * sigmahatalpha[,,5]

pal075n[,i] <- solve.QP(Dmat= Dmatrice075[,,i], dvec = devecmu075, Amat = Amatm , bvec = bvec0 , meq=1)$solution

numa075[i] <- (t(vet1) %*% invsiga075 %*% muhatalpha[,5] ) - lam[i]

eta075[i] <- numa075[i]/denoma075

}

reta075n <- array(0, c(100000))

sa075n <- array(0, c(100000))

for(i in 1:100000)

{

reta075n[i] <- t(pal075n[,i])%*%muhatalpha[,5]

sa075n[i] <- t(pal075n[,i]) %*% sigmahatalpha[,,5] %*% pal075n[,i]

}

plot(sa075n, reta075n, type="l", main= expression(paste(alpha, " = 0.75 ")), xlab ="Portfolio Variance", ylab="Portfolio Expected Return", xlim=c(0,0.004), ylim=c(0,0.04), col=3)

# alpha = 1

invsiga1 <- solve(sigmahatalpha[,,6] )

denoma1 <- t(vet1) %*% invsiga1 %*% vet1

numa1 <- array(0, c(100000))

eta1 <- array(0, c(100000))

pal1n <- array(0, c(8,100000))

Dmatrice1 <-array(0, c(8,8,100000))

devecmu1 <- muhatalpha[,6]

for(i in 1:100000)

{

Dmatrice1[,,i] <- lam[i] * sigmahatalpha[,,6]

pal1n[,i] <- solve.QP(Dmat= Dmatrice1[,,i], dvec = devecmu1, Amat = Amatm , bvec = bvec0 , meq=1)$solution

numa1[i] <- (t(vet1) %*% invsiga1 %*% muhatalpha[,6] ) - lam[i]

eta1[i] <- numa1[i]/denoma1

}

reta1n <- array(0, c(100000))

sa1n <- array(0, c(100000))

for(i in 1:100000)

{

reta1n[i] <- t(pal1n[,i])%*%muhatalpha[,6]

sa1n[i] <- t(pal1n[,i]) %*% sigmahatalpha[,,6] %*% pal1n[,i]

}

plot(sa1n, reta1n, type="l", main= expression(paste(alpha, " = 1 ")), xlab ="Portfolio Variance", ylab="Portfolio Expected Return", xlim=c(0,0.004), ylim=c(0,0.04), col=3)

# Fig.4

plot(sa0n, reta0n, type="l",main = "Short selling not allowed", lty=1, xlab ="Portfolio Variance", ylab="Portfolio Expected Return", xlim=c(0,0.004), ylim=c(0,0.04), col=2)

par(new=T)

plot(sa01n, reta01n, type="l", lty=2, xlab ="Portfolio Variance", ylab="Portfolio Expected Return", xlim=c(0,0.004), ylim=c(0,0.04), col=3)

par(new=T)

plot(sa02n, reta02n, type="l", lty= 3, xlab ="Portfolio Variance", ylab="Portfolio Expected Return", xlim=c(0,0.004), ylim=c(0,0.04), col=4)

par(new=T)

plot(sa05n, reta05n, type="l",lty=4, ylab=" ", col=5, xlim=c(0, 0.004), ylim = c(0, 0.04), xlab=" ")

par(new=T)

plot(sa075n, reta075n, type="l",lty=5, ylab=" ", col=6, xlim=c(0, 0.004), ylim = c(0, 0.04), xlab=" ")

par(new=T)

plot(sa1n, reta1n, type="l",lty=6, ylab=" ", col=1, xlim=c(0, 0.004), ylim = c(0, 0.04), xlab=" ")

legend("topright", c(expression(paste(alpha, " = 0 ")), expression(paste(alpha, " = 0.1 ")),

expression(paste(alpha, " = 0.2 ")),expression(paste(alpha, " = 0.5 ")), expression(paste(alpha, " = 0.75 ")),

expression(paste(alpha, " = 1 "))), lty=c(1:6), col=c(2,3,4,5,6,1))

Codes for Figures 6 and 7

# S-estimators

fastSloc <- function(x, N=20, k=2, best.r=5, bdp=.5, seed)

{

# A fast procedure to compute an S-estimator

# proposed by Salibian-Barrera, M. and Yohai, V.J. (2005),

# "A fast algorithm for S-regression estimates".

# This version for multivariate location/scatter was implemented by

# Kristel Joossens, K.U. Leuven, Belgium and Ella Roelant, Ghent University, Belgium.

#

# Input:

# x is the data matrix of size (n,p)

# N = number of sub-samples (default=20)

# k = number of refining iterations in each subsample (default=2)

# bdp = breakdown point (default=0.5)

# best.r = number of "best betas" to remember

# from the subsamples. These will be later

# iterated until convergence (default=5)

#

# Output is a list with components

# mean=robust estimate of location (vector: length)

# covariance=robust estimate of scatter (matrix: p,p)

# scale=value of the objective function (number)

n <- nrow(x)

p <- ncol(x)

cc = Tbsc(bdp,p)

kp = (cc/6) * Tbsb(cc,p)

if(!missing(seed)) set.seed(seed)

best.mus <- matrix(0, best.r, p)

best.sigmas <- matrix(0,best.r*p,p)

best.scales <- rep(1e20, best.r)

s.worst <- 1e20

n.ref <- 1

for(i in 1:N)

{ #

# get a subsample

#

singular <- T

while (singular==T) {

indices <- sample(n,p+1)

xs <- x[indices,]

mu <- colMeans(xs)

sigma <- cov(xs)

singular <- (det(sigma)<1e-30)

}

sigma<-(det(sigma))^(-1/p)*sigma

if (k>0) {

# do the refining

tmp <- re.s(x=x,initial.mu=mu,initial.sigma=sigma,k=k,conv=0,kp=kp,cc=cc)

mu.rw <- tmp$mu.rw

sigma.rw <- tmp$sigma.rw

scale.rw <- tmp$scale.rw

rdis.rw <- resdis(x,mu.rw,sigma.rw)

#pesi.rw <- tmp$pesi.rw

} else { #k = 0 means "no refining"

mu.rw <- mu

sigma.rw <- sigma

rdis.rw <- resdis(x,mu.rw,sigma.rw)

scale.rw <- median(abs(rdis.rw))/.6745

}

if (i > 1) {

# if this isn't the first iteration....

scale.test <- loss.S(rdis.rw,s.worst,cc)

if (scale.test < kp) {

s.best <- scale1(rdis.rw,kp,cc,scale.rw)

ind <- order(best.scales)[best.r]

best.scales[ind] <- s.best

best.mus[ind,] <- mu.rw

bm1 = (ind-1)*p;

best.sigmas[(bm1+1):(bm1+p),] <- sigma.rw

s.worst <- max(best.scales)

}

} else { # if this is the first iteration, then this is the best beta...

best.scales[best.r] <- scale1(rdis.rw,kp,cc,scale.rw)

best.mus[best.r,] <- mu.rw

bm1 = (best.r-1)*p;

best.sigmas[(bm1+1):(bm1+p),] <- sigma.rw

}

}

# do the complete refining step until convergence (conv=1) starting

# from the best subsampling candidate (possibly refined)

super.best.scale <- 1e20

# magic number alert

for (i in best.r:1) {

index = (i-1)*p;

tmp <- re.s(x=x,initial.mu=best.mus[i,],

initial.sigma=best.sigmas[(index+1):(index+p),],

initial.scale=best.scales[i],k=0,conv=1,kp=kp,cc=cc)

if (tmp$scale.rw < super.best.scale) {

super.best.scale <- tmp$scale.rw

super.best.mu <- tmp$mu.rw

super.best.sigma <- tmp$sigma.rw

#bestpesi <- tmp$pesi.rw

}

}

super.best.sigma = super.best.scale^2*super.best.sigma

return(list(mean=as.vector(super.best.mu),covariance=super.best.sigma,

scale=super.best.scale#,pesi=bestpesi

))

}

################################################################################

# the objective function, we solve loss.S(u,s,cc)=b for "s"

loss.S <- function(u,s,cc) mean(rho(u/s,cc) )

################################################################################

f.w <- function(u, cc)

{

# weight function = psi(u)/u

tmp <- (1 - (u/cc)^2)^2

tmp <- tmp * cc^2/6

tmp[ abs(u/cc) > 1 ] <- 0

return(tmp)

}

################################################################################

ksiint <- function(c,s,p) {(2^s)*gamma(s+p/2)*pgamma(c^2/2,s+p/2)/gamma(p/2)}

################################################################################

norm <- function(x) sqrt( sum( x^2 ) )

################################################################################

rho <- function(u,cc)

{

w <- abs(u)<=cc

v <- (u^2/(2)*(1-(u^2/(cc^2))+(u^4/(3*cc^4))))*w +(1-w)*(cc^2/6)

return(v)

}

################################################################################

resdis <- function(x,mu,sigma)

{

# central <- t(t(x)-mu)

# sqdis <- rowSums(((central %*% solve(sigma))*central)

central <- t(x)-mu

sqdis <- colSums(solve(sigma,central)*central)

dis <- sqdis^(0.5)

return(dis)

}

################################################################################

scale1 <- function(u, kp, cc, initial.sc=median(abs(u))/.6745)

{

# find the scale, full iterations

max.it <- 200

# magic number alert

#sc <- median(abs(u))/.6745

sc <- initial.sc

i <- 0

eps <- 1e-20

# magic number alert

err <- 1

while( ( (i <- i+1) < max.it ) && (err > eps) ) {

sc2 <- sqrt( sc^2 * mean( rho(u /sc,cc ) ) / kp )

err <- abs(sc2/sc - 1)

sc <- sc2

}

return(sc)

}

################################################################################

re.s <- function(x,initial.mu,initial.sigma,initial.scale,k,conv,kp,cc)

{

# does "k" IRWLS refining steps from "initial.beta"

#

# if "initial.scale" is present, it's used, o/w the MAD is used

# k = number of refining steps

# conv = 0 means "do k steps and don't check for convergence"

# conv = 1 means "stop when convergence is detected, or the

# maximum number of iterations is achieved"

# b and cc = tuning constants of the equation

#

n <- nrow(x)

p <- ncol(x)

rdis <- resdis(x,initial.mu,initial.sigma)

if (missing( initial.scale )) {

initial.scale <- scale <- median(abs(rdis))/.6745

} else {scale <- initial.scale}

if (conv == 1) k <- 50

# if conv == 1 then set the max no. of iterations to 50 magic number alert!!!

mu <- initial.mu

sigma <- initial.sigma

lower.bound <- median(abs(rdis))/cc

for(i in 1:k) {

# do one step of the iterations to solve for the scale

scale.super.old <- scale

#lower.bound <- median(abs(rdis))/1.56

scale <- sqrt( scale^2 * mean( rho(rdis/scale,cc) ) / kp )

# now do one step of IRWLS with the "improved scale"

weights <- f.w(rdis/scale,cc)

W <- weights %*% matrix(rep(1,p),ncol=p)

xw <- x* W/mean(weights)

mu.1 <- apply(xw,2,mean)

res <- x-matrix(rep(1,n),ncol=1)%*%mu.1

sigma.1 <- t(res)%*%((weights%*%matrix(rep(1,p),ncol=p))*(res))

sigma.1 <- (det(sigma.1))^(-1/p)*sigma.1

if (det(sigma.1)<1e-7) {

mu.1 <- initial.mu

sigma.1 <- initial.sigma

scale <- initial.scale

break

}

if (conv==1) {

# check for convergence

if ( norm( mu - mu.1) / norm(mu) < 1e-20 ) break

# magic number alert!!!

}

rdis <- resdis(x,mu.1,sigma.1)

mu <- mu.1

sigma <- sigma.1

}

rdis <- resdis(x,mu,sigma)

# get the residuals from the last beta

return(list(mu.rw = mu.1,sigma.rw=sigma.1,scale.rw = scale#, pesi.rw = W

))

}

################################################################################

Tbsb <- function(c,p)

{

y1 = ksiint(c,1,p)*3/c-ksiint(c,2,p)*3/(c^3)+ksiint(c,3,p)/(c^5);

y2 = c*(1-pchisq(c^2,p));

return(y1+y2)

}

################################################################################

Tbsc <- function(alpha,p,maxit = 1e3,eps = 1e-8,diff = 1e6)

{

# constant for Tukey Biweight S

talpha = sqrt(qchisq(1-alpha,p))

ctest = talpha

iter = 1;

while ((diff>eps)*(iter<maxit))

{

cold = ctest

ctest = Tbsb(cold,p)/alpha

diff = abs(cold-ctest)

iter = iter+1

}

return(ctest)

}

###################################################################################

############ rolling schema ######################################################

###################################################################################

library(quadprog)

library(MASS)

nsam <- 172

dN <- 8

capT <- 100

nroll <- nsam - capT + 1 # L-T+1

stepmax <- 800

dport <- read.csv(file="C:/sleoni/Aida2011/Real Data/DatiInsieme.csv", sep=";",dec=",", header=TRUE)

x <- array(0, c(nsam,dN))

x[,1]<-dport$FRANCE

x[,2]<-dport$GERMANY

x[,3]<-dport$ITALY

x[,4]<-dport$JAPAN

x[,5]<-dport$PACIFIC.EX.JAPAN

x[,6]<-dport$SPAIN

x[,7]<-dport$UNITED.KINGDOM

x[,8]<-dport$USA

alpha0 <- 0

alpha025 <-0.25

# initial values for location and scale

muinit <- apply(x,2,mean)

sigmainit <- cov(x)

muinitroll0<- array(0, c(nroll,dN))

sigmainitroll0<- array(0, c(nroll,dN,dN))

muinitroll025<- array(0, c(nroll,dN))

sigmainitroll025<- array(0, c(nroll,dN,dN))

estmu0 <- array(0, c(nroll,dN,stepmax+1))

estsig0 <- array(0, c(nroll,dN,dN,stepmax+1))

investsig0 <- array(0, c(nroll,dN,dN,stepmax+1))

pesiwj0 <- array(0, c(nroll,nsam,stepmax+1))

numpwj0 <- array(0, c(nroll,nsam, stepmax+1))

denpwj0 <- array(0, c(nroll,stepmax+1))

pesiwjxj10 <- array(0, c(nsam,dN,stepmax+1))

sigdasom0 <- array(0, c(nsam,dN,dN,stepmax+1))

estmu025 <- array(0, c(nroll,dN,stepmax+1))

estsig025 <- array(0, c(nroll,dN,dN,stepmax+1))

investsig025 <- array(0, c(nroll,dN,dN,stepmax+1))

pesiwj025 <- array(0, c(nroll,nsam,stepmax+1))

numpwj025 <- array(0, c(nroll,nsam, stepmax+1))

denpwj025 <- array(0, c(nroll,stepmax+1))

pesiwjxj1025 <- array(0, c(nsam,dN,stepmax+1))

sigdasom025 <- array(0, c(nsam,dN,dN,stepmax+1))

muhata0roll <- array(0, c(nroll,dN))

sigmahata0roll <- array(0, c(nroll,dN,dN))

muhata025roll <- array(0, c(nroll,dN))

sigmahata025roll <- array(0, c(nroll,dN,dN))

Smuhat <- array(0,c(nroll,dN))

Ssigmahat <- array(0,c(nroll,dN,dN))

Smuhat2<- array(0,c(nroll,dN))

Ssigmahat2 <- array(0,c(nroll,dN,dN))

palSminvar <- array(0, c(nroll,dN))

palSminvar25 <- array(0, c(nroll,dN))

pal0minvar <- array(0, c(nroll,dN))

pal025minvar <- array(0, c(nroll,dN))

DSmatriceminvar <-array(0, c(dN,dN,nroll))

DSmatriceminvar25 <-array(0, c(dN,dN,nroll))

palSnrminvar <- array(0, c(nroll,dN))

palSnrminvar25 <- array(0, c(nroll,dN))

Dmatriceminvar0 <-array(0, c(dN,dN,nroll))

pal0nrminvar <- array(0, c(nroll,dN))

Dmatriceminvar025 <-array(0, c(dN,dN,nroll))

pal025nrminvar <- array(0, c(nroll,dN))

datiroll <- array(0, c(nroll,capT,dN))

for(kk in 1:nroll) # rolling-horizon procedure

{

datiroll[kk,,]<-x[kk:(kk+capT-1),]

Smuhat[kk, ] <-fastSloc(datiroll[kk,,], bdp=.5)$mean

Ssigmahat[kk,,] <-fastSloc(datiroll[kk,,], bdp=.5)$covariance

Smuhat2[kk, ] <-fastSloc(datiroll[kk,,], bdp=.25)$mean

Ssigmahat2[kk,,] <-fastSloc(datiroll[kk,,], bdp=.25)$covariance

muinitroll0[kk,] <- apply(datiroll[kk,,],2,mean)

sigmainitroll0[kk,,] <- cov(datiroll[kk,,])

muinitroll025[kk,] <- apply(datiroll[kk,,],2,mean)

sigmainitroll025[kk,,] <- cov(datiroll[kk,,])

ste <- 1

estmu0[kk,,1] <- muinitroll0[kk,]

estsig0[kk,,,1] <- sigmainitroll0[kk,,]

investsig0[kk,,,1] <- solve(estsig0[kk,,,1])

estmu025[kk,,1] <- muinitroll025[kk,]

estsig025[kk,,,1] <- sigmainitroll025[kk,,]

investsig025[kk,,,1] <- solve(estsig025[kk,,,1])

ste <- 2

xjmenomu0 <- array(0, c(capT, dN))

xjmenomu025 <- array(0, c(capT, dN))

for(j in 1: capT)

{

xjmenomu0[j, ] <- datiroll[kk,j,]-estmu0[kk,,1]

xjmenomu025[j, ] <- datiroll[kk,j,]-estmu025[kk,,1]

numpwj0[kk,j,1] <- exp(-alpha0 * t(xjmenomu0[j,]) %*% (investsig0[kk,,,1]) %*% xjmenomu0[j, ]/2)

numpwj025[kk,j,1] <- exp(-alpha025 * t(xjmenomu025[j,]) %*% (investsig025[kk,,,1]) %*% xjmenomu025[j, ]/2)

}

denpwj0[kk,1] <- sum(numpwj0[kk,,1])

denpwj025[kk,1] <- sum(numpwj025[kk,,1])

for(j in 1: capT)

{

pesiwj0[kk,j,1] <- numpwj0[kk,j,1]/denpwj0[kk,1]

pesiwj025[kk,j,1] <- numpwj025[kk,j,1]/denpwj025[kk,1]

for(dd in 1:dN){

pesiwjxj10[j,dd,1] <- pesiwj0[kk,j,1]*datiroll[kk,j,dd]

pesiwjxj1025[j,dd,1] <- pesiwj025[kk,j,1]*datiroll[kk,j,dd]

}

}

for(dd in 1:dN) {

estmu0[kk,dd,2] <- sum(pesiwjxj10[,dd,1])

estmu025[kk,dd,2] <- sum(pesiwjxj1025[,dd,1])

}

for(j in 1: capT)

{

sigdasom0[j,,,2] <- (alpha0+1)*pesiwj0[kk,j,1]*(datiroll[kk,j,]-estmu0[kk,,2]) %*% t(datiroll[kk,j,]-estmu0[kk,,2])

sigdasom025[j,,,2] <- (alpha025+1)*pesiwj025[kk,j,1]*(datiroll[kk,j,]-estmu025[kk,,2]) %*% t(datiroll[kk,j,]-estmu025[kk,,2])

}

for(dd1 in 1:dN) {

for(dd2 in 1:dN) {

estsig0[kk,dd1,dd2,2] <- sum(sigdasom0[,dd1,dd2,2])

estsig025[kk,dd1,dd2,2] <- sum(sigdasom025[,dd1,dd2,2])

} }

diffmu0 <- array(1,c(dN,stepmax+1))

diffmu025 <- array(1,c(dN,stepmax+1))

for(dd in 1:dN)

{

diffmu0[dd,ste]<-abs(estmu0[kk,dd,ste]-estmu0[kk,dd,ste-1])

diffmu025[dd,ste]<-abs(estmu025[kk,dd,ste]-estmu025[kk,dd,ste-1])

}

diffsigma0 <- array(1,c(dN,dN,stepmax+1))

diffsigma025 <- array(1,c(dN,dN,stepmax+1))

for(dd1 in 1:dN) {

for(dd2 in 1:dN) {

diffsigma0[dd1, dd2, ste] <- abs(estsig0[kk,dd1,dd2,ste]-estsig0[kk,dd1,dd2,ste-1])

diffsigma025[dd1, dd2, ste] <- abs(estsig025[kk,dd1,dd2,ste]-estsig025[kk,dd1,dd2,ste-1])

}

}

while(((max(diffmu0[,ste])>0.00000000001)||(max(diffsigma0[,,ste]) >0.00000000001)||(max(diffmu025[,ste]) >0.00000000001)||(max(diffsigma025[,,ste]) >0.00000000001))&&(ste<=stepmax))

{

determestsig0 <- det(estsig0[kk,,,ste])

determestsig025 <- det(estsig025[kk,,,ste])

if (determestsig0 <= 0.00000000001) {

for(dd in 1:dN){

estsig0[kk,dd,dd,ste]<- estsig0[kk,dd,dd,ste]+ 0.0001

} }

if (determestsig025 <= 0.00000000001) {

for(dd in 1:dN){

estsig025[kk,dd,dd,ste]<- estsig025[kk,dd,dd,ste]+ 0.0001

} }

determestsig0 <- det(estsig0[kk,,,ste])

investsig0[kk,,,ste] <- solve(estsig0[kk,,,ste])

determestsig025 <- det(estsig025[kk,,,ste])

investsig025[kk,,,ste] <- solve(estsig025[kk,,,ste])

xjmenomu0 <- array(0, c(capT, dN))

xjmenomu025 <- array(0, c(capT, dN))

for(j in 1: capT)

{

xjmenomu0[j,] <- datiroll[kk,j,]-estmu0[kk,,ste]

xjmenomu025[j,] <- datiroll[kk,j,]-estmu025[kk,,ste]

numpwj0[kk,j,ste] <- exp(-alpha0* t(xjmenomu0[j,]) %*% (investsig0[kk,,,ste]) %*% xjmenomu0[j,]/2)

numpwj025[kk,j,ste] <- exp(-alpha025* t(xjmenomu025[j,]) %*% (investsig025[kk,,,ste]) %*% xjmenomu025[j,]/2)

}

denpwj0[kk,ste] <- sum(numpwj0[kk,,ste])

denpwj025[kk,ste] <- sum(numpwj025[kk,,ste])

for(j in 1: capT)

{

pesiwj0[kk,j,ste] <- numpwj0[kk,j,ste]/denpwj0[kk,ste]

pesiwj025[kk,j,ste] <- numpwj025[kk,j,ste]/denpwj025[kk,ste]

for(dd in 1:dN){

pesiwjxj10[j,dd,ste] <- pesiwj0[kk,j,ste]*datiroll[kk,j,dd]

pesiwjxj1025[j,dd,ste] <- pesiwj025[kk,j,ste]*datiroll[kk,j,dd]

}

}

ste <- ste+1

for(dd in 1:dN) {

estmu0[kk,dd,ste] <- sum(pesiwjxj10[,dd,ste-1])

estmu025[kk,dd,ste] <- sum(pesiwjxj1025[,dd,ste-1])

}

for(j in 1: capT)

{

sigdasom0[j,,,ste] <- (alpha0+1)*pesiwj0[kk,j,ste-1]* (datiroll[kk,j,]-estmu0[kk,,ste]) %*% t(datiroll[kk,j,]-estmu0[kk,,ste])

sigdasom025[j,,,ste] <- (alpha025+1)*pesiwj025[kk,j,ste-1]* (datiroll[kk,j,]-estmu025[kk,,ste]) %*% t(datiroll[kk,j,]-estmu025[kk,,ste])

}

for(dd1 in 1:dN) {

for(dd2 in 1:dN) {

estsig0[kk,dd1, dd2, ste] <- sum(sigdasom0[,dd1,dd2,ste])

estsig025[kk,dd1, dd2, ste] <- sum(sigdasom025[,dd1,dd2,ste])

}

}

for(dd in 1:dN)

{

diffmu0[dd,ste]<-abs(estmu0[kk,dd,ste]-estmu0[kk,dd, ste-1])

diffmu025[dd,ste]<-abs(estmu025[kk,dd,ste]-estmu025[kk,dd, ste-1])

}

for(dd1 in 1:dN) {

for(dd2 in 1:dN) {

diffsigma0[dd1, dd2, ste] <- abs(estsig0[kk,dd1,dd2,ste]-estsig0[kk,dd1,dd2, ste-1])

diffsigma025[dd1, dd2, ste] <- abs(estsig025[kk,dd1,dd2,ste]-estsig025[kk,dd1,dd2, ste-1])

}

}

}

muhata0roll[kk,] <- estmu0[kk,,ste]

sigmahata0roll[kk,,] <- estsig0[kk,,,ste]

muhata025roll[kk,] <- estmu025[kk,,ste]

sigmahata025roll[kk,,] <- estsig025[kk,,,ste]

invsiga0 <- solve(sigmahata0roll[kk,,])

invsiga025 <- solve(sigmahata025roll[kk,,])

invsiga1 <- solve(Ssigmahat[kk,,])

invsiga25<- solve(Ssigmahat2[kk,,])

vet1 <- array(1,dim=c(dN,1))

# short selling allowed

######################################

denomv0 <- (t(vet1) %*% invsiga0 %*% vet1)

pal0minvar[kk,] <- (invsiga0 %*% vet1 )%*%(1/denomv0)

denomv025 <- (t(vet1) %*% invsiga025 %*% vet1)

pal025minvar[kk,] <- (invsiga025 %*% vet1 )%*%(1/denomv025)

denomv <- (t(vet1) %*% invsiga1 %*% vet1)

palSminvar[kk,] <- (invsiga1 %*% vet1 )%*%(1/denomv)

denomv25 <- (t(vet1) %*% invsiga25 %*% vet1)

palSminvar25[kk,] <- (invsiga25 %*% vet1 )%*%(1/denomv25)

# short selling not allowed

######################################

Amatm <- cbind(t(array(1, dim = c(1,dN))), diag(dN))

bvec0 <- as.matrix(c(1, rep.int(0,dN)))

DSmatriceminvar[,,kk] <- Ssigmahat[kk,,]

DSmatriceminvar25[,,kk] <- Ssigmahat2[kk,,]

devecmuminvar <- array(0,c(dN))

palSnrminvar[kk,] <- solve.QP(Dmat= DSmatriceminvar[,,kk], dvec = devecmuminvar, Amat = Amatm , bvec = bvec0 , meq=1)$solution

palSnrminvar25[kk,] <- solve.QP(Dmat= DSmatriceminvar25[,,kk], dvec = devecmuminvar, Amat = Amatm , bvec = bvec0 , meq=1)$solution

Dmatriceminvar0[,,kk] <- sigmahata0roll[kk,,]

pal0nrminvar[kk,] <- solve.QP(Dmat= Dmatriceminvar0[,,kk], dvec = devecmuminvar, Amat = Amatm , bvec = bvec0 , meq=1)$solution

Dmatriceminvar025[,,kk] <- sigmahata025roll[kk,,]

pal025nrminvar[kk,] <- solve.QP(Dmat= Dmatriceminvar025[,,kk], dvec = devecmuminvar, Amat = Amatm , bvec = bvec0 , meq=1)$solution

}

# Short selling allowed, Fig.6

par(mfrow = c(2, 2))

boxplot(pal0minvar[,], ylim=c(-0.7,1.2), xlab="Asset number", ylab="Weights", main=expression(paste("Min-var using MLE")) )

boxplot(pal025minvar[,], ylim=c(-0.7,1.2), xlab="Asset number", ylab="Weights", main=expression(paste("Min-var using MPE with ",alpha==0.25)) )

boxplot(palSminvar25[,], ylim=c(-0.7,1.2), xlab="Asset number", ylab="Weights", main=expression(paste("Min-var using S-estimators with ",epsilon^"*"==0.25)) )

boxplot(palSminvar[,], ylim=c(-0.7,1.2), xlab="Asset number", ylab="Weights", main=expression(paste("Min-var using S-estimators with ",epsilon^"*"==0.5)) )

# Short selling not allowed, Fig.7

par(mfrow = c(2, 2))

boxplot(pal0nrminvar[,], ylim=c(0,1), xlab="Asset number", ylab="Weights", main=expression(paste("Min-var using MLE")) )

boxplot(pal025nrminvar[,], ylim=c(0,1), xlab="Asset number", ylab="Weights", main=expression(paste("Min-var using MPE with ",alpha==0.25)) )

boxplot(palSnrminvar25[,], ylim=c(0,1), xlab="Asset number", ylab="Weights", main=expression(paste("Min-var using S-estimators with ",epsilon^"*"==0.25)) )

boxplot(palSnrminvar[,], ylim=c(0,1), xlab="Asset number", ylab="Weights", main=expression(paste("Min-var using S-estimators with ",epsilon^"*"==0.5)) )
